# Supplementary material for: MXene-driven nanoscale field-effect junction for advanced 4-terminal perovskite/silicon tandem solar panels
Source: Nat Commun. 2026 Mar 6;17:3394. doi: 10.1038/s41467-026-70002-4 (PMC13065735; doi:10.1038/s41467-026-70002-4)
Supplement: Supplementary file 1 — Supplementary Information [file 41467_2026_70002_MOESM1_ESM.pdf]

Supplementary Information

MXene-driven nanoscale field-effect junction for advanced 4-terminal perovskite/silicon tandem solar panels

A. Agresti,[1§*] S. Pescetelli,[1§*] G. Viskadouros,[2] A. Pazniak[3], E. Leonardi,[4] A. Di Vito,[1] P. Amiri,[1] M. Auf Der Maur,[1] F. Menchini,[5] S. Del Gobbo,[5] F. Di Giacomo,[1] G. Bengasi,[6] C. Connelli,[6] L. Sorbello,[4] M. Foti,[6] F. Bonaccorso,[7] E. Kymakis,[2,8] A. Di Carlo[1,9*]

[1] CHOSE, Centre for Hybrid and Organic Solar Cells, University of Rome Tor Vergata, via del Politecnico 1, 00133 Roma, Italy

[2] Department of Electrical and Computer Engineering, Hellenic Mediterranean University, Heraklion, Greece

Greece.

[3] Université Grenoble Alpes, CNRS, Grenoble INP, LMGP, F-38000, Grenoble, France.

[4] Halocell Europe - Viale Castro Pretorio 122, 00185 Rome, Italy.

[5] Energy Technologies and Renewable Sources Department,ENEA, C.R. Casaccia, Via Anguillarese, 301 00123 Roma, - Italy.

[6] 3SUN - Enel Green Power (EGP) SpA, Contrada Blocco Torrazze - 95121 Catania, Italy.

[7] BeDimensional S.p.A., Lungotorrente Secca, 30R, 16163 Genova, Italy.

[8] Institute of Emerging Technologies, Hellenic Mediterranean University Research Center, Heraklion, Crete.

[9] Istituto di Struttura della Materia, CNR-ISM, via del fosso del cavaliere 100, 00133 Roma, Italy

§ Both authors equally contributed to the work

*Corresponding authors:

Agresti Antonio: antonio.agresti@uniroma2.it

Pescetelli Sara: sara.pescetelli@uniroma2.it

Di Carlo Aldo: aldo.dicarlo@uniroma2.it

Author Orcid:

Agresti Antonio 0000-0001-6581-0387

Pescetelli Sara 0000-0002-3336-2425

Di Carlo Aldo 0000-0001-6828-2380

SECTION S.I. 1 Optimization perovskite recipe in term of band gap.

The optimization of the perovskite recipe in term of energy band gap has been achieved by tuning the composition of the perovskite precursor solution and eventually the film stoichiometry. Two different perovskite formulas have been tested moving the band gap from 1.65 eV for the triple-cation formulation to 1.68 eV in the case of double-cation one, as evident by the Tauc plot reported Supplementary Fig. 1.

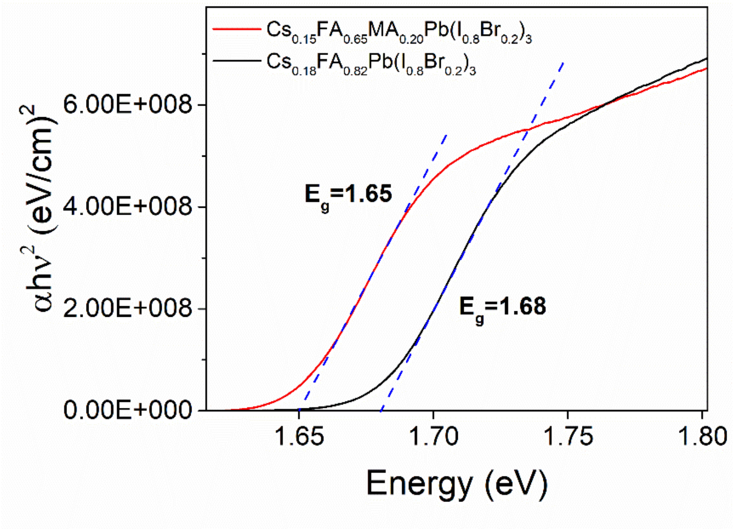

Supplementary Fig. 1: **Optical bandgap tuning of perovskite formulations.** Tauc plots for the two tested perovskite formulations. The extracted values are 1.65 eV and 1.68 eV for $Cs_{0.15}FA_{0.65}MA_{0.20}Pb(I_{0.8}Br_{0.2})_3$ and $Cs_{0.18}FA_{0.82}Pb(I_{0.8}Br_{0.2})_3$ films on glass, respectively.

SECTION S.I. 2 Optimization of MX-Cl concentration for 1 ml of perovskite solution.

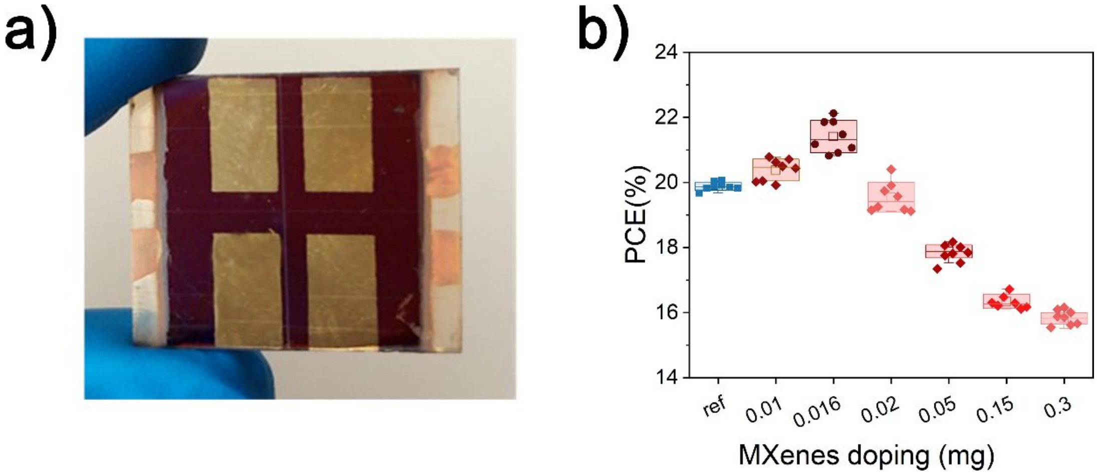

Supplementary Fig. 2: Optimization of MXene-Cl concentration in perovskite precursor solution. a) Photograph of one of the two glass substrates used for device fabrication, each hosting four small-area perovskite solar cells. A total of 12 devices were fabricated for the statistical evaluation of photovoltaic parameters (**Fig. 2,**) while 8 devices were used for the optimization of the MXene-Cl doping concentration, **b)** PCE of PSCs realized by varying the amount of MXene-Cl added to 1 ml of perovskite precursor solution.

SECTION S.I. 3 Characterization of Cl based Mxene

After Lewis acid molten salt etching, multilayered $Ti_3C_2Cl_2$ MXenes were delaminated and drop-casted onto a Si/SiO_2 substrate to evaluate their quality. XRD data show the complete conversion of Ti_3AlC_2 to $Ti_3C_2Cl_2$ with a characteristic shift of 00*l* diffraction peaks towards lower angles indicating an increase in c-lattice parameter to 22.34 Å (Supplementary Fig. 3).

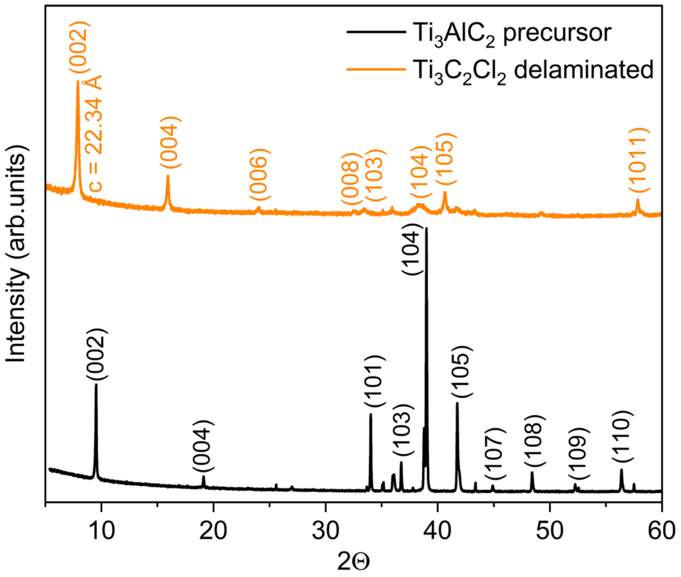

Supplementary Fig. 3: Structural conversion from Ti_3AlC_2 MAX phase to $Ti_3C_2Cl_2$ MXene. XRD of the Ti_3AlC_2 MAX phase precursor (black curve) and delaminated $Ti_3C_2Cl_2$ (orange curve).

When using molten salt etching, the presence of a uniform chalcogen or halogen termination of the outer transition metal in the MXene structure is assumed,[1] which differs from the randomly distributed mixed surface groups in the case of MXene etching in F-based aqueous solutions.[2] To access the chemical composition of the $Ti_3C_2Cl_2$ MXenes after synthesis and delamination, we performed SEM/EDS, the results of which are shown in Supplementary Fig. 4. The EDS data confirm that the surface of Ti_3C_2 is covered predominantly by Cl groups with a Ti/Cl ratio of 1.59 and a small amount of incorporated O after synthesis (4.07 at. %) and delamination (5.47 at. %).

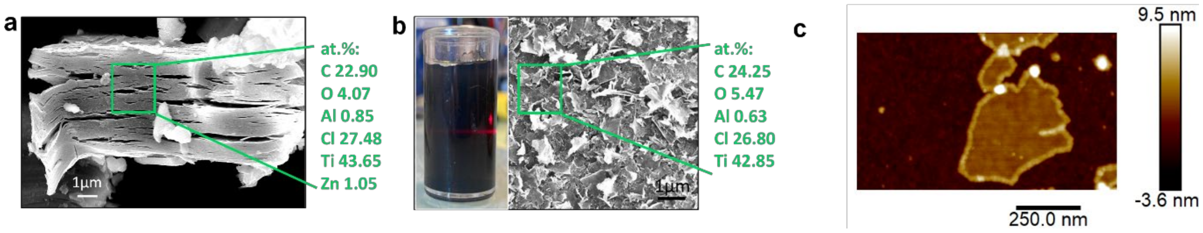

Supplementary Fig. 4: Morphology and elemental composition of Cl-terminated Ti_3C_2 MXenes. SEM images with corresponding chemical composition of **a)** multilayer $Ti_3C_2Cl_2$ and **b)** delaminated $Ti_3C_2Cl_2$; **c)** AFM topography of the Cl-terminated Ti_3C_2 MXene flakes.

Details about the surface chemistry of $Ti_3C_2Cl_2$ were extracted by XPS measurements presented in Supplementary Fig. 5.

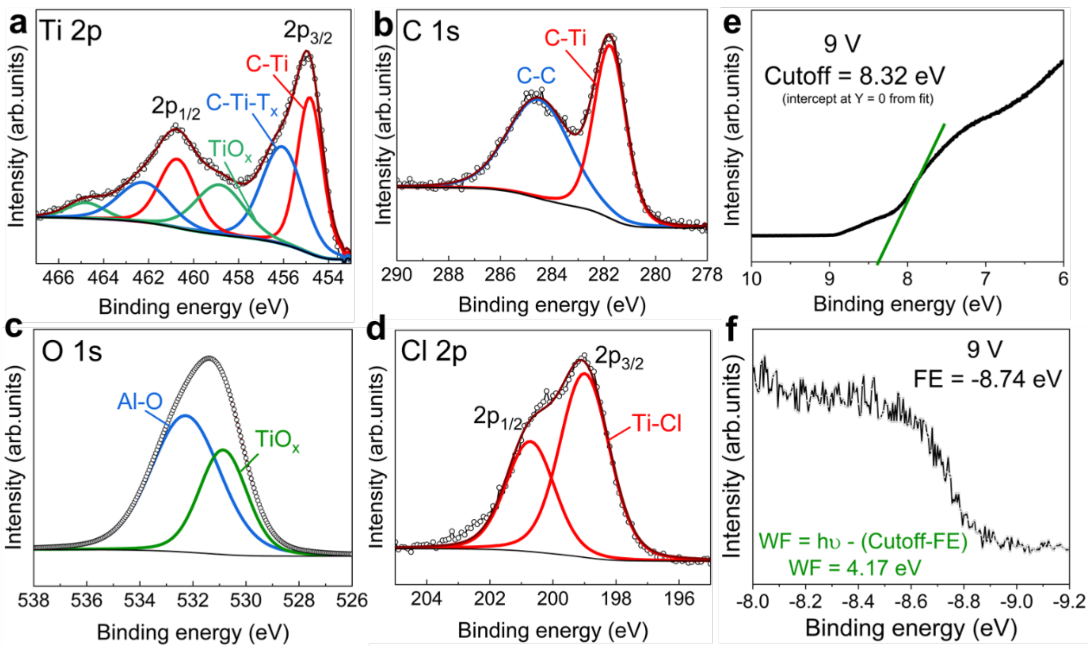

Supplementary Fig. 5: Electronic structure of $Ti_3C_2Cl_2$ MXenes probed by XPS and UPS. MXene characterization: **a-d)** HR XPS spectra of $Ti_3C_2Cl_2$ MXenes; **e-f)** UPS spectra of the valence band region of $Ti_3C_2Cl_2$ MXenes.

The *Ti 2p* region (Supplementary Fig. 5a) consists of three major components: Ti-C (the binding energy position of the 3/2 spin orbit component is 454.9 eV), C-Ti-T_x, corresponding to the termination of Ti atoms of a higher valence predominantly with Cl surface groups (456.1 eV) and a minor portion of Ti oxide (458.9 eV). The *C 1s* region (Supplementary Fig. 5b) consists of the Ti-C component (281.8 eV) from the Ti-C MXene skeleton and C-C component (284.7 eV) originating from amorphous and graphitic carbon formed as the result of chemical exfoliation and delamination. The *O 1s* region (Supplementary Fig. 5c) has two peaks located at 530.8 eV and 532.3 eV and correspond to Ti oxide and Al-O bonds. The peaks at 198.9 eV and 200.6 eV at *Cl 2p* region (Supplementary Fig. 5d) correspond to Cl-Ti bonds[3] and confirm the Cl termination of Ti_3C_2 MXenes. The electronic property of $Ti_3C_2Cl_2$ was featured by work function (WF) measurement using UPS (Supplementary Figs. 5 e-f). We found that the WF of delaminated $Ti_3C_2Cl_2$ is 4.17 eV. In addition, there is a finite density of states at the Fermi level (Supplementary Fig. 6a) suggesting metal-like behavior of $Ti_3C_2Cl_2$ MXenes.

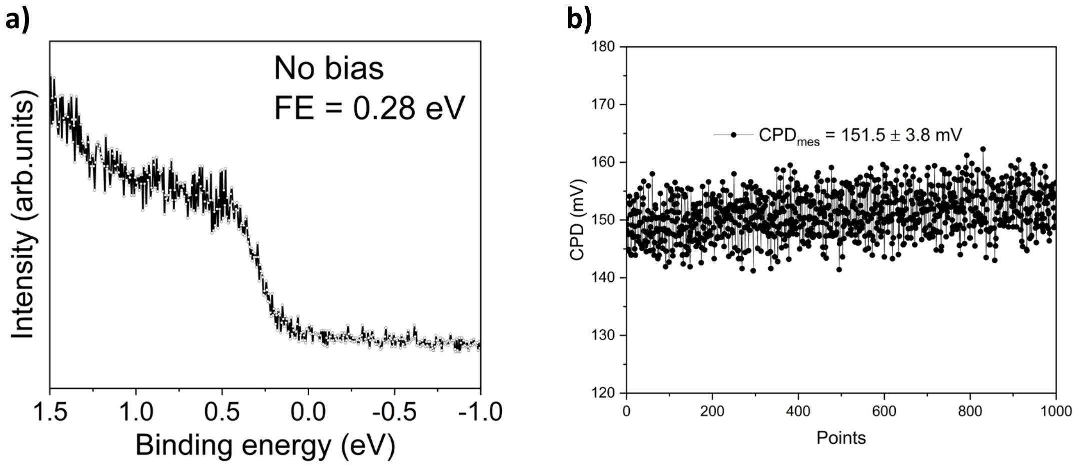

Supplementary Fig. 6: Work function determination of $Ti_3C_2Cl_2$ MXenes by UPS and Kelvin probe a) UPS spectra of the valence band region of $Ti_3C_2Cl_2$ Mxene; **b)** CPD between a test Au sample and a tip measured at 5 different points.

The Kelvin probe measures the contact potential difference (CPD) between a sample and a tip – not the WF of the sample. The WF of the sample can be calculated by determining the WF of the tip calibrated against a known surface. A test Au sample was used to determine the WF of the tip. The average value of CPD between a test Au sample and a tip measured at 5 different points is plotted in Supplementary Fig. 6.

The WF of tip can be calculated as follows:

$$WF_{tip} = WF_{Au} - CPD_{mes}$$

$$WF_{tip} = 5100 - 151.5 = 4948.5 \text{ meV} = 4.95 \text{ eV}$$

After that, the samples were measured at several points and the WF of the sample is calculated as follows:

$$WF_{sam} = WF_{tip} + CPD_{mes}$$

SECTION S.I. 4 Density of Current-Voltage (J-V) for the opaquely investigated cell typologies and Maximum Power Point (MPP) tracking for the best-performing opaque cells employing the 2D material-engineered structure (G/MX/4-FPEAI) vs reference (G/EA).

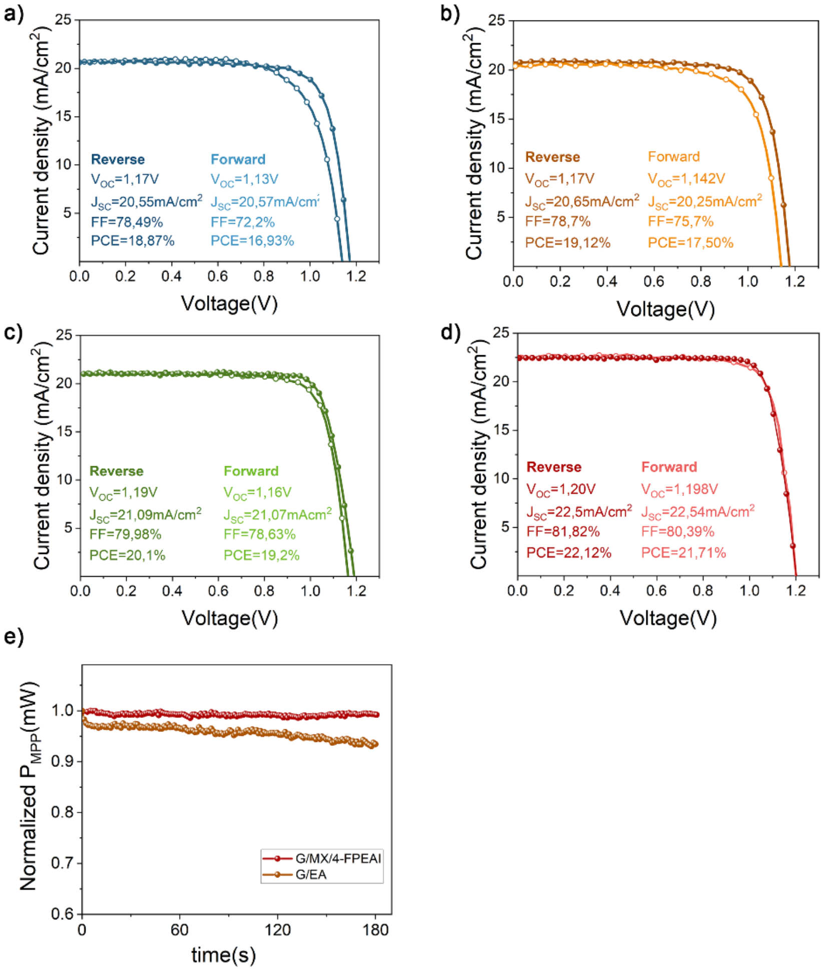

Supplementary Fig. 7: J–V hysteresis and maximum power point tracking of opaque perovskite solar cells. Density of Current-Voltage (J-V) characteristics, acquired in forward and reverse voltage scan directions for the best efficient **a)** G/CB, **b)** G/EA, **c)** G/4-FPEAI, **d)** G/MX/4-FPEAI cells. **e)** Normalized Maximum Power Point (MPP) tracking for the best-performing opaque cell employing the 2D material-engineered structure (G/MX/4-FPEAI) vs reference structure (G/EA).

From Supplementary Figs. 7 a), b), c), d), we extracted the forward and reverse scan values of V_{OC}, FF, J_{SC} and PCE for each of the four opaque PSC structures. As shown by the data, the difference in open-circuit voltage between reverse and forward scan (ΔV_{OC}) ranges from 0.04 V in the G/CB reference device down to 0.002 V

in the optimized G/MX/4-FPEAI device. Similarly, the fill factor difference (ΔFF) decreases from 6.3% in G/CB to 1.4% in G/MX/4-FPEAI. This clearly demonstrates that the combined MXene and 4-FPEAI strategy significantly reduces hysteresis, in terms of PCE between forward and reverse scan directions. The ΔPCE decreases from 1.94% in G/CB devices to just 0.4% in G/MX/4-FPEAI.
This trend supports the conclusion that our dual strategy (SGP + MX-Cl) effectively mitigates interfacial recombination and ionic migration, leading to more stable and symmetric device performance under J–V scanning.
Supplementary Fig. 7e) Further corroborates these findings, showing the normalized, stabilized P_{MPP} curves under MPPT conditions for the G/EA and G/MX/4-FPEAI devices. The G/MX/4-FPEAI cell not only maintains a higher steady-state output but also exhibits improved long-term operational stability, highlighting the role of surface gradient passivation and MXene doping in suppressing degradation pathways under continuous illumination.

SECTION S.I. 5 Characterization of perovskite films: Scan Electron Microscopy (SEM) image, time-resolved photoluminescence (TRPL) measurements, transient photo-voltage (TPV) measurements, Incident Photon to current Conversion Efficiency (IPCE) measurements, UV-Vis Spectroscopy and Photoluminescence spectroscopy (PL).

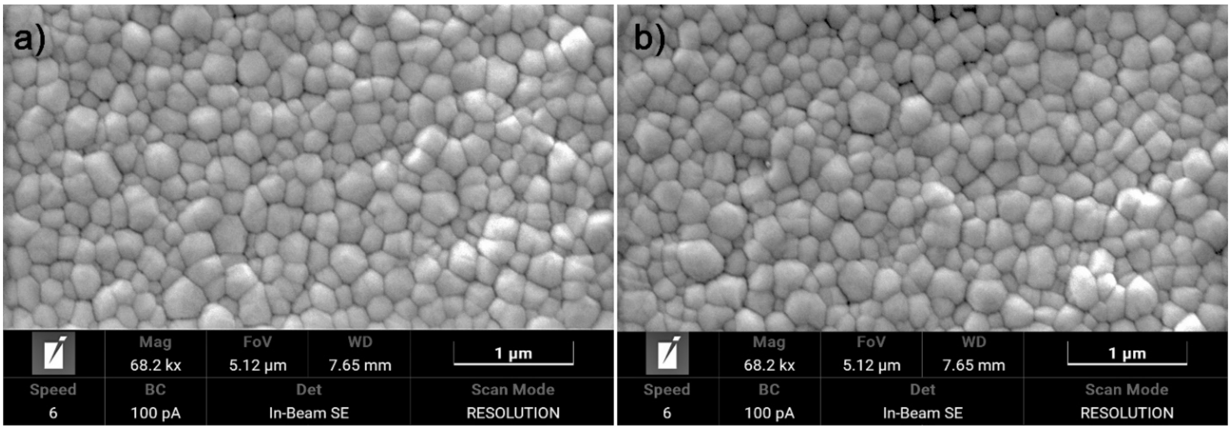

Supplementary Fig. 8: Surface morphology of perovskite films processed with different anti-solvents. SEM image of perovskite film surface obtained by applying **a)** CB and **b)** EA as anti-solvent in the second step of perovskite film fabrication.

To further elucidate the impact of MX-Cl on the crystallinity of perovskite film, XRD measurements were performed on 4-FPEAI and MX-Cl/4-FPEAI perovskite films and are reported in **Fig. 3e** in the main text**.** The lattice planes of cubic perovskite structure's (110), (220), and (310) can be identified by the peaks observed in all perovskite films, which are at around 14.10°, 28.38°, and 31.80°, respectively. The addition of MX-Cl resulted in a stronger intensity of the perovskite diffraction peaks, suggesting that MX-Cl can enhance the crystallinity of the perovskite film.[4] Furthermore, the crystal structure of the perovskite films did not change after MX-Cl were incorporated, as evidenced by no impurity peaks appearing in the XRD patterns of MX-Cl/4-FPEAI films.

Supplementary Tab. 1: **Peak XRD analysis**. Peak intensity ratio of (110)/(310) and (220)/(310) calculated from corresponding XRD patterns for the perovskite films with and without MX-Cl

	**(110)/(310)**	**(220)/(310)**
**4-FPEAI**	1.62	0.92
**MX-Cl/4-FPEAI**	2.29	1.31

Moreover, we determined the peak intensity ratios of (110)/(310) and (220)/(310) reported in Supplementary Tab. 1 and observed they exhibited a similar trend to the peak intensities of (110) and (220). These results suggest that the introduction of MX-Cl not only enhances the crystallinity of the perovskite film but also influences the grain alignment, guiding a preferred orientation during the crystallization process.[5] As a result, the improved crystallinity of the perovskite film is effective in reducing the lattice distortion of perovskite film and results in a lessening of defect density and trap states.[5–8] Furthermore, the presence of fewer trap states in the perovskite modified by MX-Cl/4-FPEAI compared to the 4-FPEAI based devices, could be imputed to the strong interaction between the Cl terminations of MX-Cl and Pb^{2+} ions leading to a reduction of non-radiative recombination introduced by uncoordinated metallic Pb. In addition, these interactions could be contributing to a decreased residual tensile strain by giving rise to a more resilient perovskite lattice.
To further evaluate the influence of Cl-functionalized MXenes on the optoelectronic properties of the perovskite films, we performed time-resolved photoluminescence (TRPL) measurements. The decay profiles of the reference and MXene-treated films were fitted using a bi-exponential decay model:

$$F(t) = A_1 e^{-t/\tau_1} + A_2 e^{-t/\tau_2}$$

where τ_1 and τ_2 represent the fast and slow decay lifetimes, respectively, and A_1, A_2 are the corresponding amplitudes. The fast decay component (τ_1) is commonly associated with trap-assisted recombination processes, while the slow component (τ_2) reflects radiative recombination of free carriers.
As shown in Supplementary Tab. 2, the MXene-treated film shows a longer average carrier lifetime (τ_{avg} = 111.5 ns) compared to the reference (68.76 ns). The fast decay time τ_1 also increases from 41.63 ns to 75.67 ns, indicating fewer shallow trap states. This reduction is likely due to larger domain sizes induced by MXene flakes acting as localized crystallization templates, which decrease the overall grain boundary density and associated trap sites. The amplitude A_1 also increases significantly (from 3.16 to 9.127), further suggesting a dominant role of non-radiative pathways in the control film that are effectively passivated by the MXene layer.
These findings corroborate the beneficial role of Cl-functionalized MXenes in reducing trap-assisted recombination by passivating defect sites—likely through interaction between Cl^- terminations and undercoordinated Pb^{2+} ions. The TRPL results align with structural improvements observed via XRD and SEM, further confirming that the incorporation of MXenes enhances the film's crystallinity, reduces trap densities, and improves charge carrier dynamics.

Supplementary Tab. 2: Fitting parameters extracted from the bi-exponential fitting of TRPL decay curves for the reference and MX-Cl modified perovskite films. A_1 and A_2 are the amplitudes of the fast and slow decay components, respectively; τ_1 corresponds to trap-assisted (non-radiative) recombination, and τ_2 to radiative recombination. τ_{avg} is the average carrier lifetime calculated as $\tau_{avg}=(A_1\tau_1^2+A_2\tau_2^2)/(A_1\tau_1+A_2\tau_2)$. The increased τ_1 and τ_{avg} in the MXene-modified film suggest reduced trap density and suppressed non-radiative recombination.

Sample	A_1	τ_1 (ns)	A_2	τ_2 (ns)	τ_{avg} (ns)
**4-FPEAI**	3.617	41.63	0.157	199.3	68.76
**MX-Cl/4-FPEAI**	9.127	75.67	1.327	203.2	111.5

With the aim to give further inside the role of MX-doping and SGP treatment, we performed transient photo-voltage (TPV) measurements on the investigated devices by extracting the charge life-time trends, as reported in Supplementary Fig. 9a.

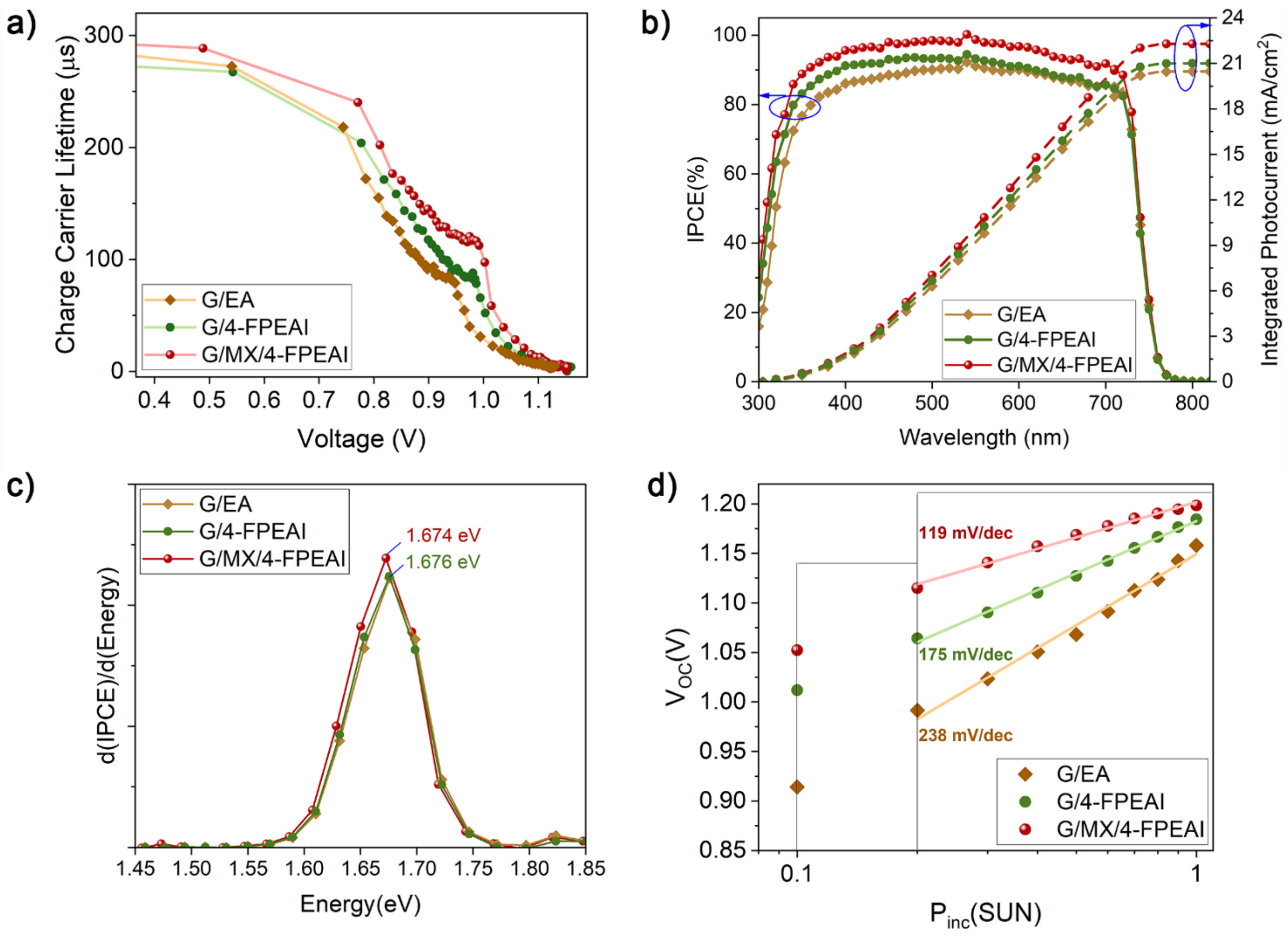

Supplementary Fig. 9. Charge recombination dynamics and optoelectronic characterization of devices a) Charge carrier lifetime extracted by the small-signal transient photo-voltage (TPV) decay profiles; **b)** Incident Photon to current Conversion Efficiency (IPCE) spectra with the integrated photocurrent density (Integrated J_{SC}) related to the best efficient cell for each proposed device structure. **c)** Derivative d(IPCE)/d(E) curves for G/EA, G/4-FPEAI, and G/MX/4-FPEAI devices. The energy position of the maximum corresponds to the optical bandgap ($E_{g,opt}$). All samples exhibit nearly identical peak energies (≈1.67–1.68 eV), confirming that neither MX-Cl nor 4-FPEAI induces measurable changes in the perovskite bandgap. **d)** V_{OC} light intensity-dependence [$V_{OC}(P_{inc})$] of the investigated PSC structures. Linear fitting of the curves has been performed for P_{inc} > 0.2 SUN, and the respective slope values are reported on the plot.

Since during the measurement no charge is extracted from the device, V_{OC} decaying transient is due to the internal recombination in the perovskite bulk and/or at the interfaces with selective contacts.[9] Notably G/MX/4-FPEAI structure showed a major enlargement in the recombination life-time with respect to the compared to the G/EA sample while the enlargement obtained in the case of G/4-FPEAI was less pronounced. On one side, this suggests that, despite the main effect of SGP consisting in inducing a p-type surface dipole at the top perovskite interface, leading to field-effect passivation and modified charge distribution, improving the device J_{SC}. Moreover, as showed in Supplementary Fig. 9b, J_{SC} rises from 21.01 mA/cm² (4-FPEAI) to 22.3 mA/cm² (G/MX/4-FPEAI), with calculated (from the integration of EQE spectra with the AM1.5G solar irradiance spectrum) and measured (under the AM1.5G reference spectrum) J_{SC} values closely matching. To verify that these recombination improvements are not associated with bandgap variations, we additionally extracted the optical bandgap ($E_{g,opt}$) of the G/EA, G/4-FPEAI and G/MX/4-FPEAI devices from the derivative of the IPCE spectra, following the methodology proposed by Carron et al.[10] (Thin Solid Films 669, 482–486, 2019). In this approach, the energy position of the maximum in the d(IPCE)/d(E) curve corresponds to the optical bandgap, provided that a single, well-defined peak is present and interference fringes are negligible. As shown in Supplementary Fig. 9c, all samples exhibit nearly identical peak energies ($E_{g,opt}$ ≈ 1.67–1.68 eV), indicating that neither the 4-FPEAI surface treatment nor the MX-Cl incorporation produces any measurable

change in the optical bandgap of the perovskite absorber. This confirms that the electronic modifications observed by UPS do not arise from variations in the band-edge positions.
Using these $E_{g,opt}$ values, the conduction band minimum (CBM) was then estimated as:

$$\text{CBM} \approx \text{VBM}_{\text{UPS}} + E_{\text{g,opt}}.$$

The corresponding CBM levels have been included in the updated energy-level diagrams in Supplementary Fig. 11. Since the CBM values remain essentially unchanged across all sample configurations, the observed work-function shifts must arise from Fermi-level shift (i.e., changes in interfacial charge density) rather than modifications of the fundamental bandgap. This validates the interpretation provided in the main text and supports the formation of a dipole-induced interfacial band bending.
Given the absence of measurable bandgap changes, the influence of MX-Cl on structural quality and recombination pathways was subsequently examined. Indeed, MX-doping is strongly effective in reducing the defect states density thanks to an enlargement in perovskite domains where MXenes are present, by reducing the impact of the grain boundaries that are well known to insert deep trap state levels in the perovskite gap. The enlarged perovskite domain size translates in a slight shift of the perovskite band gap from 1.68 eV for perovskite films with SGP (named 4-FPEAI) to 1.667 in case of same film but with the addition of MX-Cl (named MX-Cl/4-FPEAI), as reported in Supplementary Fig. 10a (measurements carried out on samples made ad-hoc for UPS measurements, see S.I. SECTION S.I. 6). Moreover, the beneficial role of MX-Cl in reducing the charge recombination is confirmed by the steady state photoluminescence (PL) measurements performed on the same 4-FPEAI and MX-Cl/4-FPEAI films (Supplementary Fig. 10b). Regarding PL spectra, generally a reduced PL emission is imputed to the non-radiative recombination acting in the film, while the presence of a red-shift emission can be ascribed to an enlargement of the averaged perovskite domain size.[11] Both phenomena can be observed in Supplementary Fig. 10b where in the case of optimized MX-Cl/4-FPEAI a higher PL emission intensity (+75.1%) is recorded together with a slightly red shift of the emission peak (763 nm) once compared with 4-FPEAI device (758nm). The demonstrated suppression of non-radiative recombination in the photo absorber layer is in line with the results carried out by TPV measurements (Supplementary Fig. 9a). The significantly reduction of trap states density in the MX-Cl/4-FPEAI film, can confidently justify the superior electrical performance showed by the G/MX/4-FPEAI devices (reported in main text in Fig. 2) in term of V_{OC} and FF.
Dark J–V measurements (Supplementary Fig. 10c) further confirm the reduced non-radiative recombination in G/MX/4-FPEAI devices. The lower reverse leakage current and reduced ideality factor compared to G/4-FPEAI are consistent with the longer TRPL lifetimes (main text, Fig. 3h) and higher PL intensity (Supplementary Fig. 10b), while the enhanced forward current indicates more efficient electron injection at the buried perovskite/m-TiO_2 interface, arising from the locally strengthened n-type character induced by MX-Cl.

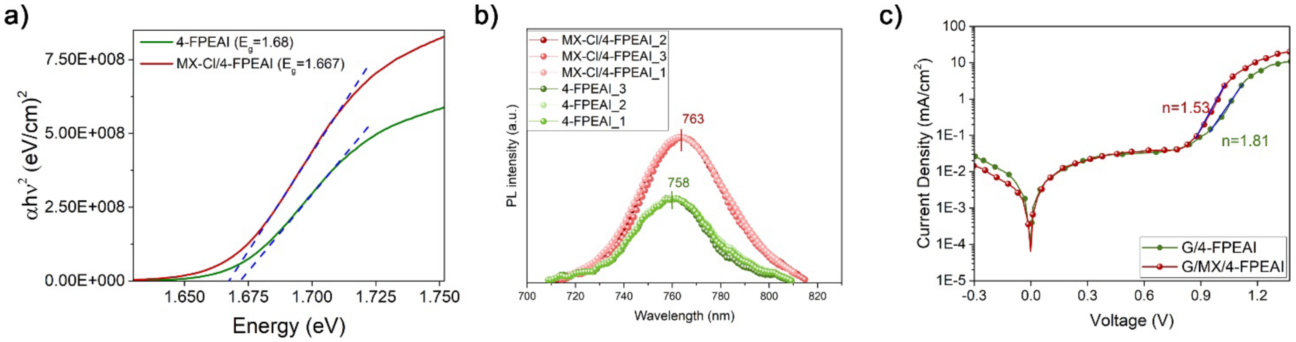

Supplementary Fig. 10: Optical and electrical characterization of MX-Cl-modified perovskite films a) Tauc plots and **b)** steady-state PL spectra for perovskite films with SGP (named 4-FPEAI) and same film but with the addition of MX-Cl (named MX-Cl/4-FPEAI). PL spectra were acquired on three different points of the samples (named _1,_2,_3); **c)** Dark current density–voltage (J-V) curves comparing G/4-FPEAI and G/MX/4-FPEAI opaque devices. The ideally factor (n) is extracted from the diffusion-dominated current region according to the equation: $n = \frac{kT}{q}\left(\frac{d(lnJ)}{dV}\right)$.

SECTION S.I. 6 Ultraviolet photoelectron spectroscopy (UPS) measurements and Kelvin probe force microscopy (KPFM) measurements.

Ultraviolet photoelectron spectroscopy (UPS) measurements

To gain insight into the energetic effects induced by the surface gradient passivation and MXene doping engineering strategies, we performed ultraviolet photoelectron spectroscopy (UPS) measurements on a set of ad-hoc fabricated perovskite thin films with reduced thickness (comparable to the MXene flake size, ~200 nm) and varied interfacial treatments (REF, MX-Cl, 4-FPEAI, and MX-Cl/4-FPEAI). The extracted values of work function (WF) and valence band maximum (VBM) for each cell configuration are summarized in Tab. 2 of the main text.
As shown in Supplementary Fig. 11 i), the energy level diagram reconstructed from the UPS data clearly reveals a systematic shift in both WF and VBM across the series.
The reference (REF) sample exhibits a WF of 4.75 eV and a VBM located 0.9 eV below the Fermi level (EF), corresponding to an ionization energy (IE) of 5.65 eV. Upon incorporation of $Ti_3C_2Cl_2$ MXenes (MX-Cl), the WF decreases to 4.55 eV while the VBM remains unchanged at 0.9 eV. This behavior indicates that the addition of MXenes enhances the localized n-type character of the perovskite, consistent with the formation of a surface dipole at the perovskite/MXene interface together with the introduction of donor states near perovskite conduction band edge (see DFT calculation reported in Supplementary Fig. 15). Notably, this interface dipole shifts the vacuum level without affecting the perovskite band structure. Conversely, the use of 4-FPEAI as SGP induces a WF increase to 4.85 eV and a shift in the VBM to 0.7 eV below EF. These results suggest a clear modification of the perovskite electronic structure, indicating a p-type interfacial electronic shift driven by surface dipole formation. The combined MX-Cl/4-FPEAI treatment results in intermediate values (WF = 4.70 eV, VBM = 0.8 eV), consistent with interfacial band bending induced by spatially separated surface dipoles at the top and buried interfaces.
These findings are corroborated by DFT simulations, which show a vacuum level shift of –0.39 eV for the MXene interface and +0.14 eV for 4-FPEAI, supporting the observed WF modulation. Overall, the UPS data confirm the formation of dipole-induced band bending localized at the two perovskite interfaces, driven by the two treatments acting at opposite interfaces.

Notably, Supplementary Fig. 12 panel a) shows that the *O 1s* XPS spectra of the pristine perovskite, 4-FPEAI-treated, and MX-Cl/4-FPEAI samples exhibit nearly identical contributions from adsorbed water (82.2%, 84.0%, and 86.0%, respectively). These values lie well within the intrinsic ±3–4% variability of multi-component *O 1s* peak fitting, indicating that neither MX-Cl nor 4-FPEAI significantly alter the hydrophilicity of the perovskite surface. This confirms that the energy-level shifts observed in UPS/XPS arise from the intended interfacial chemical modifications, at the buried m-TiO_2/perovskite interface and at the top perovskite surface, rather than from differences in environmental exposure or water uptake.

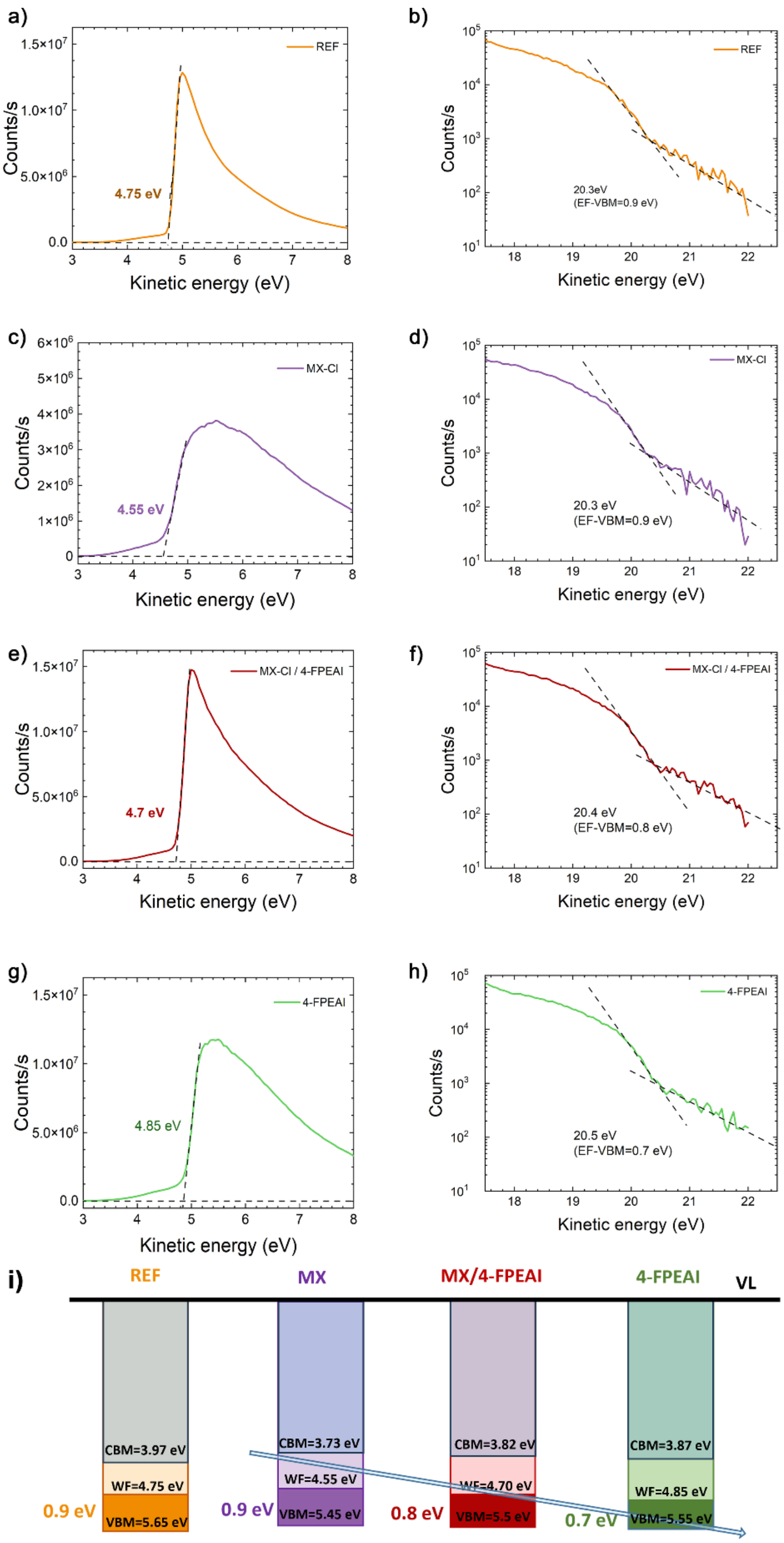

Supplementary Fig. 11. Energy level alignment of perovskite films with interfacial treatments. UPS spectra for perovskite films modified with different interface strategies: reference (**a–b**), MX-Cl (**c–d**), MX-Cl/4-FPEAI (**e–f**), and 4-FPEAI (**g–h**). Panels (**a, c, e, g**) show the secondary electron cutoff region used to extract the WF, while panels (**b, d, f, h**) display the valence band region used to determine the VBM with respect to the EF. A gradual modulation of the WF and VBM is observed across the series, highlighting the impact of dipole formation and band structure tuning induced by MXene doping and surface gradient passivation; **i)** Schematic energy level alignment of perovskite films extracted from UPS measurements for the four configurations: REF (untreated), MX-Cl, 4-FPEAI, and combined MX-Cl/4-FPEAI. The WF, CBM and VBM positions are plotted relative to the vacuum level. A progressive modulation of the energy levels is observed, with MX-Cl inducing a downward shift in WF (consistent with dipole-induced vacuum level bending) and 4-FPEAI causing an upward shift in both WF and VBM. The intermediate energy level alignment observed under the combined treatment suggests the formation of a field-effect junction across the perovskite layer, driven by interfacial dipoles, which promotes band bending and enhances charge extraction.

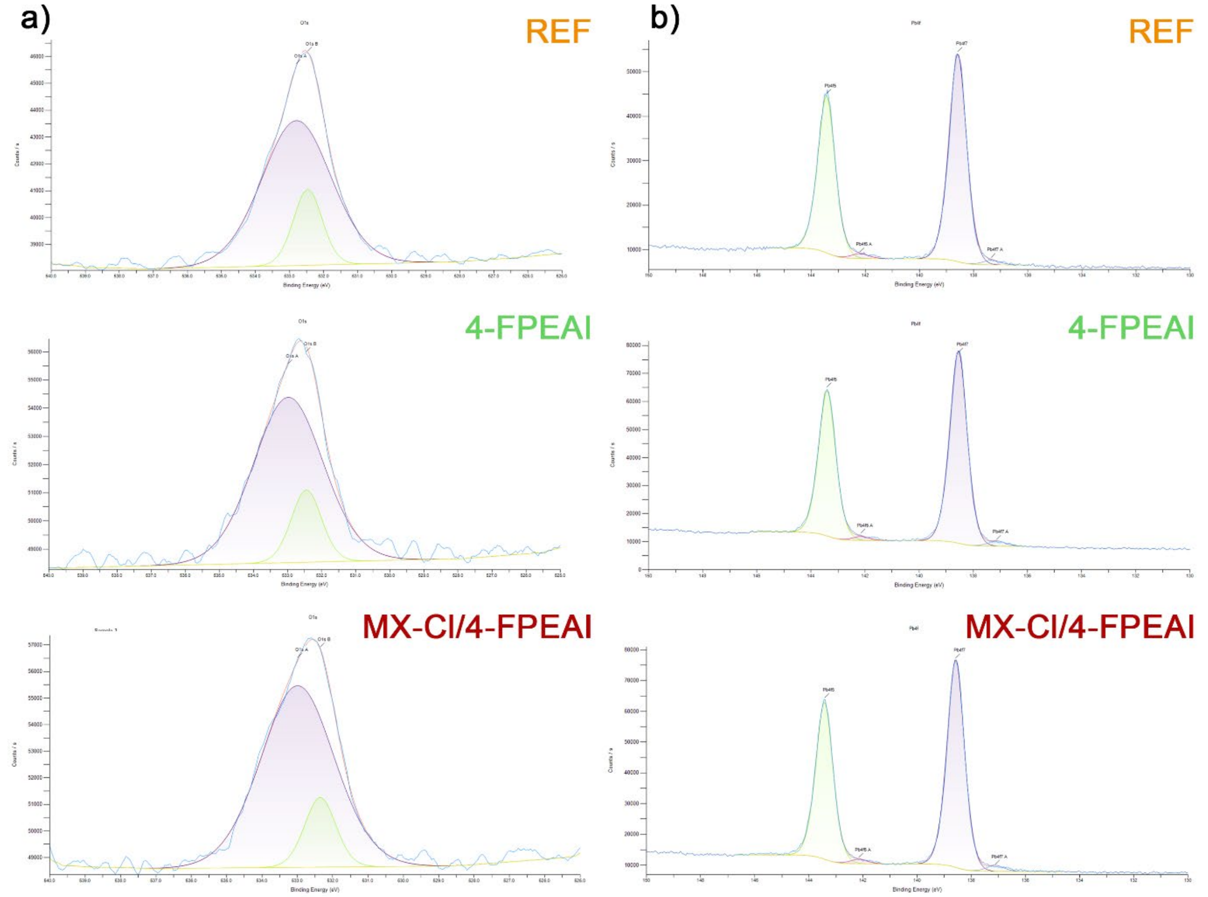

Sample	Adsorbed water	C=O	PB in PSK	Pb in PO
	Position (eV) = /relative amount			
	Panel a)		Panel b)	
**REF**	532.80/82.2%	532.47/17.8%	138.58-143.43 / 97.8%	137.30-142.15 / 2.2%
**4-FPEAI**	532.98/84.0%	532.45/16.0%	138.55-143.40 /97.3%	137.14-142.15 / 2.7%
**MX-Cl/4-FPEAI**	532.99/86.0%	532.35/14.0%	138.57-143.42 / 97.2%	137.12-142.16 / 2.8%

Supplementary Fig. 12: XPS analysis of surface chemistry in modified perovskite films. Panel **a)** XPS measurements *O1s* peaks and panel **b)** *Pb 4f* core-level spectra for reference perovskite film, 4-FPEAI treated perovskite film, 4-FPEAI treated perovskite film with MX-Cl. The samples are fabricated with a reduced thickness around 200 nm grown on $mTiO_2$.

The XPS *Pb 4f* core-level spectra of all samples show in Supplementary Fig.12 panel b) (REF, MX-Cl, 4-FPEAI, and MX-Cl/4-FPEAI) exhibit nearly identical binding energies and relative component ratios (Pb in perovskite ≈ 97–98%, Pb in PbO ≈ 2–3%), with no evidence of chemical shifts or peak broadening. This invariance confirms that the observed UPS valence-band and work-function shifts are not affected by charging, surface deterioration, or measurement artefacts, but rather arise from genuine electronic modifications induced by the different strategies: doping with MX-Cl and interfacial treatment with 4-FPEAI.

Kelvin probe force microscopy (KPFM) measurements

The same WF trends was confirmed also with KPFM performed on the same typology of ad-hoc made samples.

Supplementary Tab. 3: **Work function and contact potential difference of perovskite films with MX-Cl and surface gradient passivation**. Calculated WF and relative contact potential difference (CPD) of the investigated perovskite over FTO/G+cTiO_2/G+mTiO_2 substrate: a reference perovskite (REF), a perovskite doped with MX-Cl (MX-Cl), a perovskite including SGP (4-FPEAI), a perovskite including both SGP MX-Cl doping (MX-Cl/4-FPEAI).

	**REF**	**4-FPEAI**	**MX-Cl**	**MX-Cl/4-FPEAI**
**CPD, mV**	-188.2 ± 6.3	-150.7 ± 5.2	-214.3 ± 3.5	-161.5 ± 5.3
**WF, eV**	4.76	4.79	4.74	4.78

Since WF of MX-Cl is 4.19 eV (Supplementary Fig. 5f), the MX-Cl doping of perovskite enhances its localized n-type character, as confirmed by decreased WF value of MX-Cl versus REF sample (see Supplementary Tab. 3). Indeed, the MXene addition into the perovskite film translates in the formation of a dipole at perovskite/MXene interface, without altering the perovskite bandgap, while shifting the perovskite WF.[12] Conversely, the use of 4-FPEAI as SGP strategy gives a gradual and more evident p-type character with a WF increase up to 4.78 eV for the MX-CL/4-FPEAI.[13] Considering that MX-Cl sits at the buried interface while KPFM provide a measurement of top interface, could be concluded that the combination of 4-FPEAI and MX-Cl doping induces spatially separated interfacial dipoles at the top and buried interfaces, resulting in a field-effect junction that enhances V_{OC}, as observed for both G/4-FPEAI and G/MX/4-FPEAI cell structures (Fig. 2a).[14]

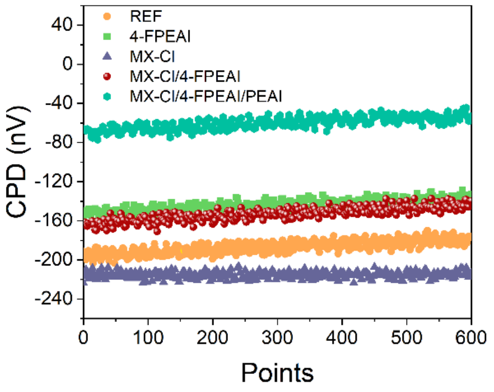

Supplementary Fig. 13: Contact potential difference of perovskite films measured by KPFM. Contact potential difference (CPD) between different samples and a tip measured at 5 different points.

SECTION S.I. 7 DFT simulations

The perovskite materials involved in the manufacturing of solar cells are usually double or triple cation perovskites. Moreover, iodine atoms are often substituted, for a certain percentage, by Br ions. A realistic simulation of such systems would require the use of very large supercells composed of hundreds of atoms, that would require a huge number of computational resources. Thus, the single cation $MAPbI_3$ perovskite is generally benchmarked in DFT simulations for the sake of feasibility. We can see in Supplementary Fig. 14 that the valence and conduction band edges of $MAPbI_3$ contributed from the iodine and lead states, while the cation states are far from the band edges, that supports the assumption, largely documented in our previous literature, that different cations behave similarly. Moreover, it has been experimentally demonstrated there that MXene doping induces a WF shift for $MAPbI_3$ perovskite as well as for triple cation perovskites.[12]

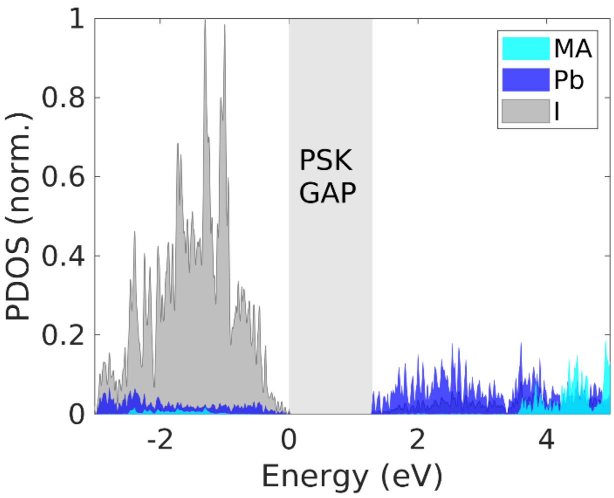

Supplementary Fig. 14: Electronic density of states of pristine $MAPbI_3$ perovskite. PDOS of pristine $MAPbI_3$ perovskite, the band gap is represented by the shaded light gray rectangle.

Supplementary Fig. 15 shows the results obtained by DFT simulations for the perovskite/MX-Cl and the perovskite/4-FPEAI interface. The Pb ions on the perovskite surface interacting with additives are highlighted by shaded red areas in Supplementary Fig. 15a and Supplementary Fig. 15d for MX-Cl and 4-FPEAI, respectively. The induced WF shift is pointed out by the red arrow in Supplementary Fig. 15b and Supplementary Fig. 15e for MX-Cl and 4-FPEAI, respectively. Note that the actual system has a small amount of MX-Cl additive in the perovskite layer, so the measured WF reduction is smaller than the calculated one. Additionally, the WF increase from 4-FPEAI is smaller than the WF decrease from MX-Cl. The PDOS for perovskite/MX-Cl and perovskite/4-FPEAI is shown in Supplementary Figs. 15c and 15f**,** respectively, where the shaded gray rectangle represents the undoped perovskite band gap, and the states contributed from the Pb ions on the perovskite surface interacting with additives are shown in red in the PDOS.

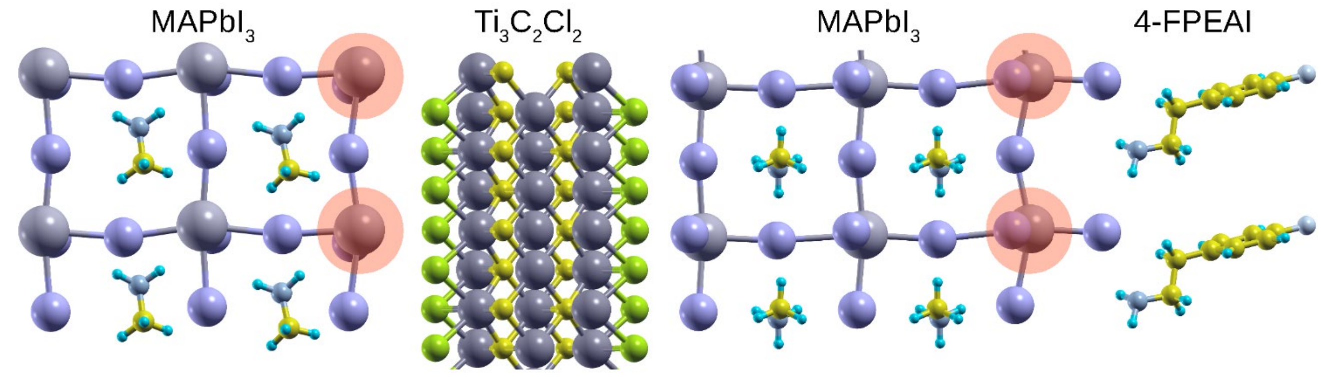

a) d)

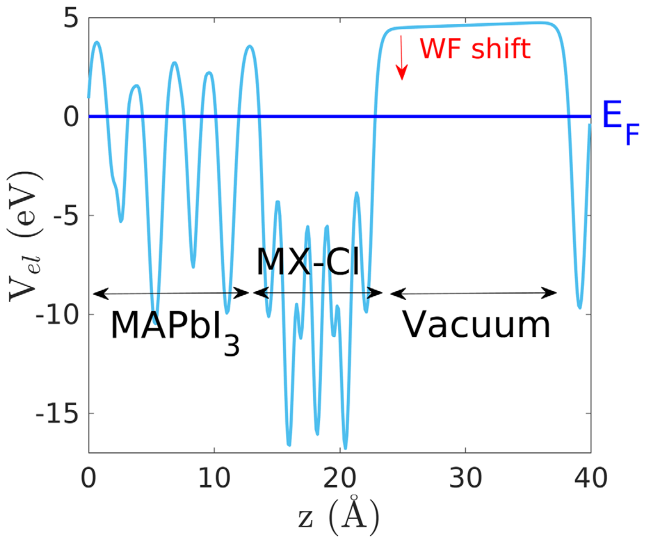

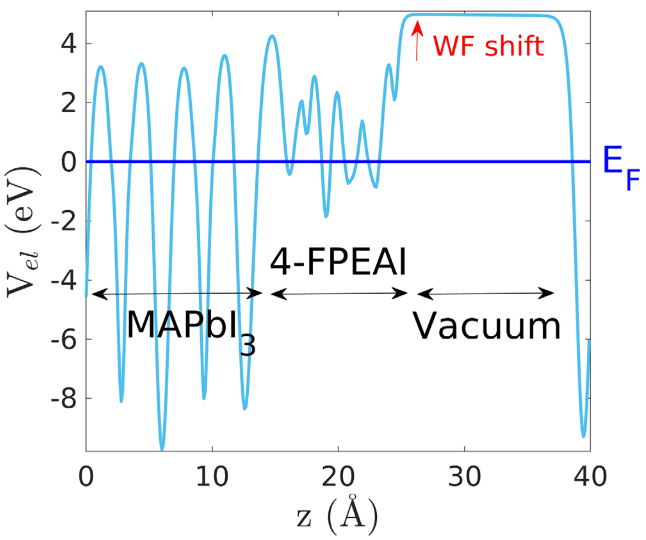

b)

e)

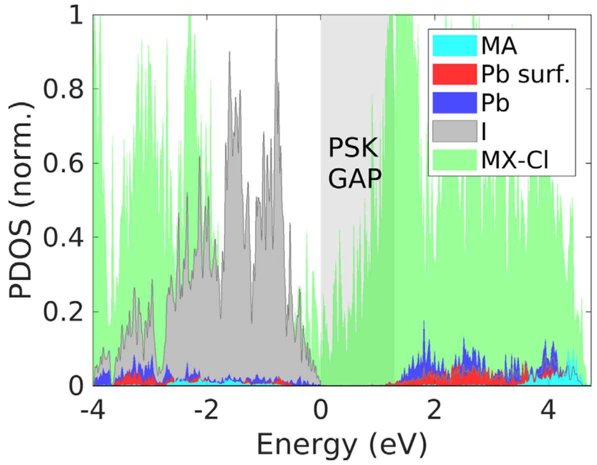

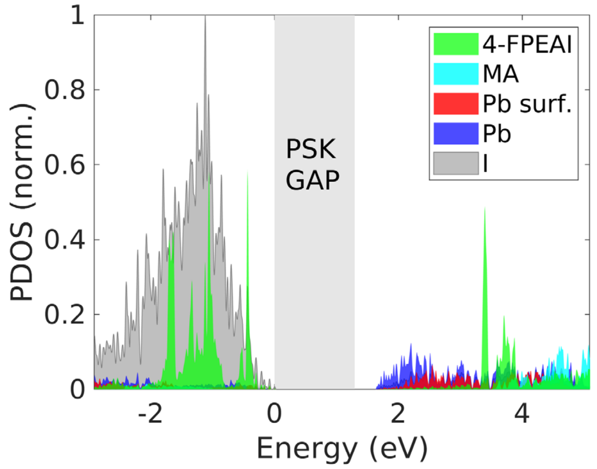

c)

f)

Supplementary Fig. 15: DFT modelling of perovskite/MXene and perovskite/4-FPEAI interfaces. a) Computed structure of the $MAPbI_3/Ti_3C_2Cl_2$ interface, the Pb atom at the PbI_2 perovskite surface interacting with MX-Cl is highlighted by the shaded red area. **b)** Electrostatic potential profile, averaged over planes, in the direction perpendicular to the MX-Cl/$MAPbI_3$/4-FPEAI interfaces. The Fermi energy is set to zero so that the vacuum potential just away from the MX-Cl and 4-FPEAI surfaces corresponds to the WF of the MX-Cl/$MAPbI_3$ and $MAPbI_3$/4-FPEAI interfaces, respectively. **c)** Projected density of states of the $MAPbI_3/Ti_3C_2Cl_2$ interacting system, the energy gap of pristine perovskite is reported by the shaded light gray rectangle. **d)** Computed structure of the $MAPbI_3$/4-FPEAI interface, the Pb atom at the PbI_2 perovskite surface interacting with 4-FPEAI is highlighted by the shaded red area. **e)** Electrostatic potential averaged over planes perpendicular to the $MAPbI_3$/4-FPEAI interface, the Fermi energy is set to zero so that the vacuum potential just away from the 4-FPEAI corresponds to the WF of the $MAPbI_3$/4-FPEAI interface. **f)** PDOS of the $MAPbI_3$/4-FPEAI interacting system, the energy gap of pristine perovskite is reported by the shaded light gray rectangle.

Concerning the substitution of iodine with bromine, we can say here that, even if the gap of $MAPbBr_3$ perovskite is higher than the one of $MAPbI_3$, the two systems behave similarly, as shown in Supplementary Fig. 16. The WF values obtained by DFT simulations for the considered systems are listed in Supplementary Tab. 4.

The simulations have been performed using the Quantum Espresso software package[12] with Perdew-Burke-Ernzerhof (PBE),[13] projector-augmented-wave (PAW), and scalar relativistic pseudo-potentials available in the Quantum Espresso pseudo-potentials library. To simulate the interaction between perovskite and additives, we employed a 6.2 Å x 6.2 Å x 40 Å tetragonal supercell, where the perovskite slab is composed of 1 x 1 x 2 pseudo-cubic unit cells exposing the PbI_2 surface. The electrostatic potential profile of the MX-Cl/$MAPbI_3$/4-FPEAI system is obtained simulating a 6.2 Å x 6.2 Å x 70 Å tetragonal supercell, where the perovskite slab is composed of 1 x 1 x 4 pseudo-cubic unit cells exposing the PbI_2 surface. The cutoff energy for the

wavefunctions was set to 50 Ry, with a k-points grid of 6 x 6 x 1. The total energy convergence threshold and the forces convergence threshold for structural optimization were set to 10^{-3} Ry and 10^{-3} Ry/Bohr, respectively.

Supplementary Tab. 4: WF values obtained by DFT simulations for the systems considered.

Simulated system	WF value (eV)
$Ti_3C_2Cl_2$	4.47
$MAPbI_3$	4.84
$MAPbI_3$ + $Ti_3C_2Cl_2$	4.45 (-0.39 with respect to pristine $MAPbI_3$)
$MAPbI_3$ + 4-FPEAI	4.98 (+0.14 with respect to pristine $MAPbI_3$)
$MAPbBr_3$	4.75
$MAPbBr_3$ + $Ti_3C_2Cl_2$	4.34 (-0.41 with respect to pristine $MAPbBr_3$)

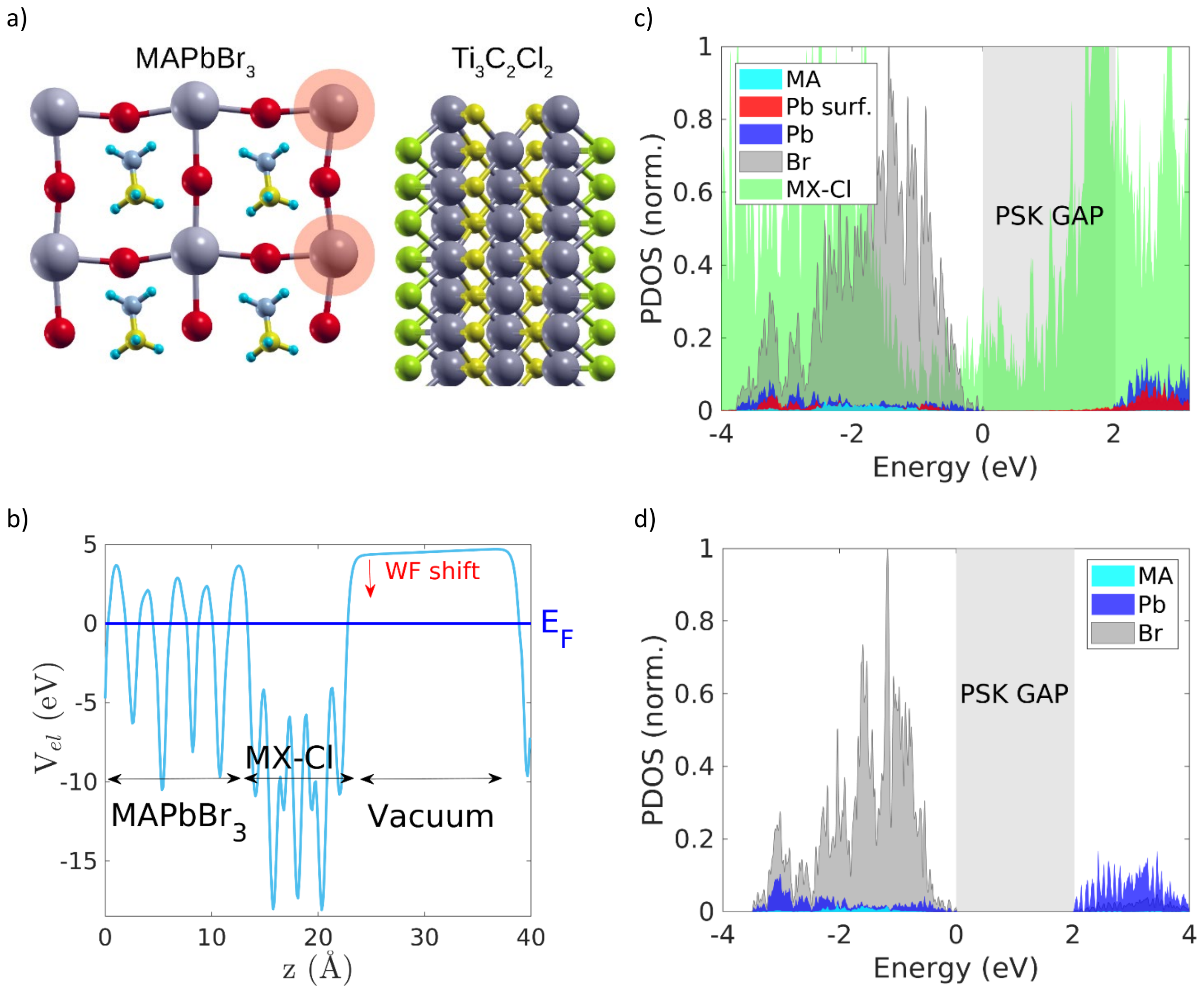

Supplementary Fig. 16: Electronic structure of $MAPbBr_3$ and $MAPbBr_3$/MXene interface from DFT. a) Computed structure of the $MAPbBr_3/Ti_3C_2Cl_2$ interface, the Pb atom at the $PbBr_2$ perovskite surface interacting with MX-Cl is highlighted by the shaded red area. **b)** Electrostatic potential averaged over planes perpendicular to the $MAPbBr_3/Ti_3C_2Cl_2$ interface, the Fermi energy is set to zero so that the vacuum potential just away from the MX-Cl surface corresponds to the WF of the $MAPbBr_3/Ti_3C_2Cl_2$ interface. **c)** Projected density of states of the $MAPbBr_3/Ti_3C_2Cl_2$ interacting system, the energy gap of pristine perovskite is reported by the shaded light gray rectangle. **d)** PDOS of pristine $MAPbBr_3$ perovskite, the band gap is represented by the shaded light gray rectangle.

SECTION S.I. 8 Device simulations

The device simulations have been performed using the TiberCAD multiphysics/multiscale simulator, and the materials database therein.[14] We used transfer matrix method for the optical part (TMM), and Poisson/drift-diffusion (DD) equations for the electrical properties (TMM/DD simulations). The most relevant parameters employed in the simulation of the reference perovskite solar cell are listed in Supplementary Tab. 5. In the second simulation setup, related to the addition of 4-FPEAI, a p-type doping profile has been considered within the perovskite layer, with maximum doping density of 5 x 10^{16} cm^{-3} at the p-side perovskite interface and an exponential decay towards the opposite side of the perovskite layer, as shown by the dashed red line in Supplementary Fig. 17. These effective doping profiles are used solely as a numerical approximation to reproduce the electrostatic effects of interfacial dipoles. Finally, the third simulation setup, related to the further addition of MX-Cl, includes also a donor type doping profile, with maximum density of 5 x 10^{16} cm^{-3} at the n-side perovskite interface and an exponential decay towards the opposite side of the perovskite layer, as depicted by the dashed magenta line in Supplementary Fig. 17. Moreover, in the third setup, the characteristic time for SRH recombination in perovskite, *i.e.*, τ_{SRH} = 60 x 10^{-9} s, is higher than the reference value, and the perovskite electron mobility for this final setup is 7.5 $cm^2V^{-1}s^{-1}$.

Supplementary Tab. 5: **List of the relevant parameters employed in TMM/DD simulation of the reference device**.

**Reference**	**c-TiO_2**	**m-TiO_2**	**perovskite**	**PTAA**
doping dens. (cm^{-3})	1e16 donor	1e16 donor	none	1e17 acceptor
CB edge (eV)	-4.00	-4.10	-3.95	-3.00
VB edge (eV)	-7.20	-7.12	-5.65	-5.45
elec. mob. ($cm^2V^{-1}s^{-1}$)	0.3	0.3	4.9	1e-8
hole mob. ($cm^2V^{-1}s^{-1}$)	0.01	0.01	4.9	1e-4
SRH rec. time (s)			20e-9	
interf. rec. velocity ($s^{-1}cm$)			40	

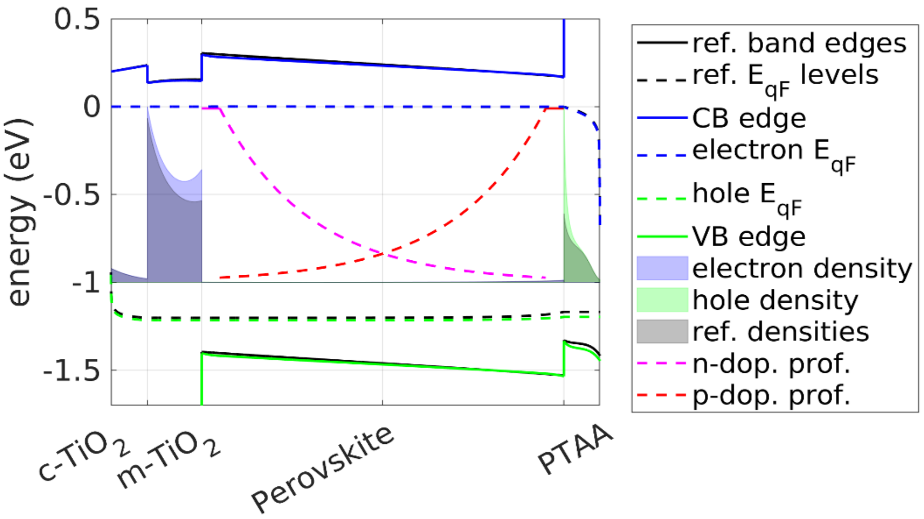

Supplementary Fig. 17: Simulated energy band profiles of optimized perovskite solar cells. Energy band profiles and quasi-Fermi levels for the optimized structure, *i.e.*, perovskite plus both additives, compared to reference, at open circuit conditions. The area plots represent the normalized carriers' densities of the optimized structure compared to reference (note that also the reference densities are normalized to the maximum values of the optimized structure, to show the relative improvement). Finally, the normalized doping profiles are shown.

SECTION S.I. 9 Structures, photovoltaic parameters and stability test of the ST-PSCs

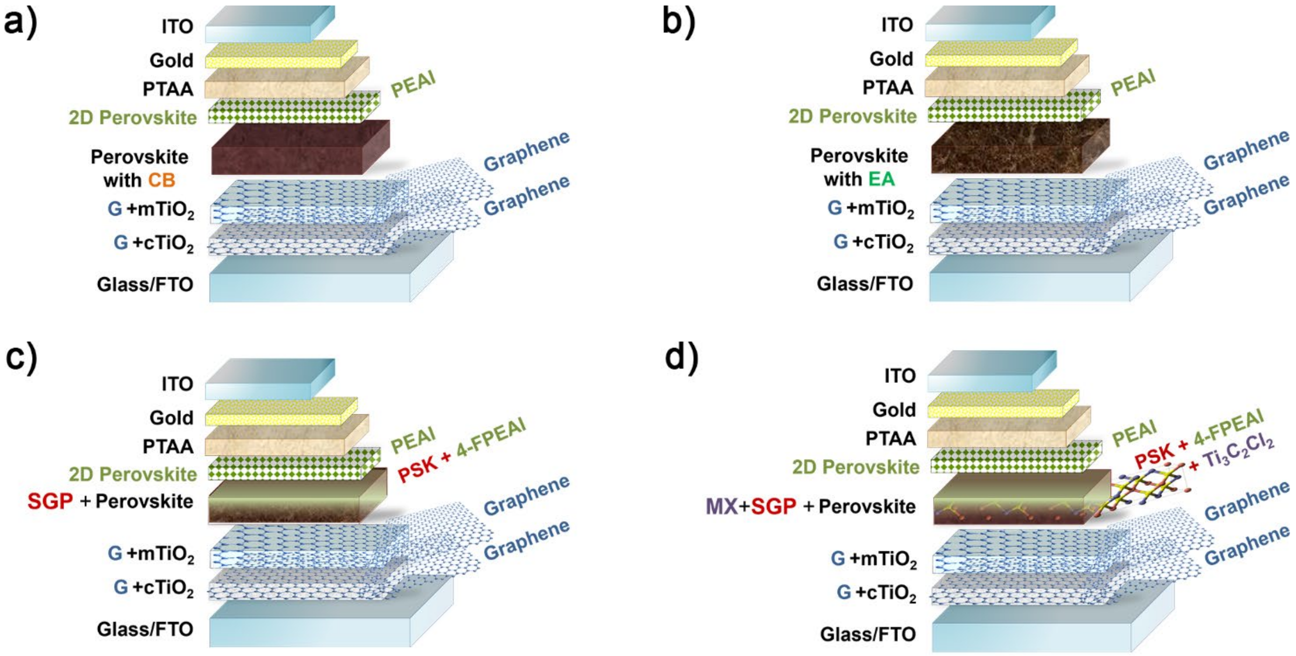

Supplementary Fig. 18: Architectures of semi-transparent perovskite solar cells. Four tested mesoporous solar cell structures for semi-transparent (ST) devices: **a)** and **b)** represent the reference structure realized employing graphene (G) within the ETL and by using of chlorobenzene-CB (panel a) or ethyl acetate-EA (panel b) as anti-solvent during the perovskite layer deposition; **c)** optimized structure realized with graphene based ETL, EA as green solvent for perovskite and surface gradient passivation-SGP strategy based on 4-FPEAI passivation to deposit the perovskite layer; **d)** complete 2D materials engineered structure using G-modified ETL, EA as green anti-solvent for perovskite with SGP strategy based on 4-FPEAI passivation and chlorine-based MXenes (MX-Cl) as dopant for perovskite layer.

Supplementary Tab. 6: **Electrical photovoltaic parameters for the four investigated ST-PSCs structures.** The values are extracted by the I-V characteristics acquired under 1 SUN irradiation in reverse scan treated as averaged values ± standard error obtained on 12 samples for each cell typology and for the best performing devices.

Device		V_{OC}(V)	J_{SC}(mA/cm^2)	FF(%)	PCE(%)
**CB**	Champion	1.191	19.5	74.04	16.77
	Average	1.179±0.0019	18.81±0.11	72.5±0.33	16.09±0.10
**EA**	Champion	1.199	19.42	74.45	17.15
	Average	1.191±0.0017	19.01±0.092	72.93±0.28	16.50±0.10
**4-FPEAI**	Champion	1.21	20.42	74.4	17.60
	Average	1.196±0.003	19.54±0.10	73.55±0.16	17.19±0.04
**MX_4-FPEAI**	Champion	1.222	20.51	75.47	18.29
	Average	1.203±0.002	19.92±0.08	73.60±0.29	17.63±0.067

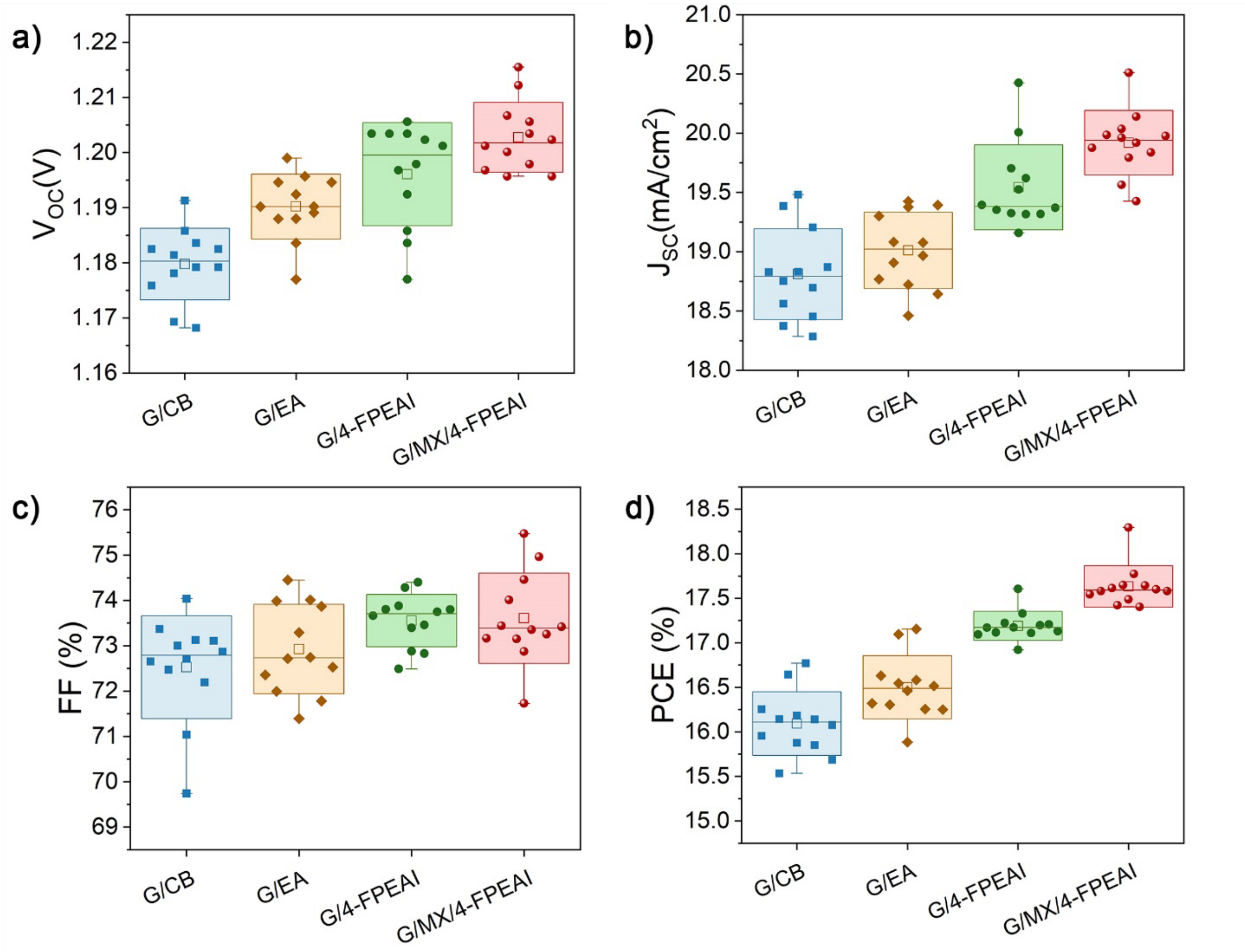

Supplementary Fig. 19: Statistical distribution of photovoltaic parameters for ST devices. Electrical parameter statistics (12 samples) for the four investigated ST-PSC structures extracted by the current-voltage (I-V) characteristics acquired under 1 SUN irradiation.

To further understand the observed variation in V_{OC} between reference and optimized ST devices, we provide here an extended discussion based on simulation results and interface analysis. The Supplementary Fig. 19 shows the statistical distribution of the main photovoltaic parameters, highlighting a reduced ΔV_{OC} improvement in ST structures compared to opaque ones. In the following, we examine possible physical origins of this behaviour, including the effect of the top contact work function, unintentional doping of the PTAA layer during ITO deposition, and the role of interfacial engineering. The analysis supports the hypothesis that the reduced ΔV_{OC} is not due to a diminished effectiveness of the MX-Cl/4-FPEAI treatment, but rather to changes induced by the device architecture and processing conditions.

Effect of the work function: due to the use of Au/ITO ST contact instead of Au, the effective work function (WF) of the anode could be slightly lower for the ST cell with respect to the opaque cell. To investigate this effect, we repeated the simulations for the reference and optimized devices varying only the effective WF value employed for the p-contact, namely, WF = 5.2 eV for the opaque cell and WF = 5.18 eV for the ST structure. The results are shown in Supplementary Fig. 20a), where the cyan solid and dashed lines represent respectively the J-V characteristics of the opaque reference and optimized devices, while the red solid and dashed lines depict the J-V curves of the ST reference and optimized devices, respectively. We can see that the role of the work function is reflected by the fill factor (FF) features, giving a smaller FF when a smaller WF is considered, while J_{SC} and V_{OC} are not affected.

Effect of an unintentional doping: a possible effect induced by the sputtering process employed to deposit the ITO layer at the p-contact is an unintentional doping of the PTAA hole transport layer (HTL). We analysed the impact of such unintentional doping comparing the simulation results of the reference and optimized

devices with the behavior obtained for the same structures but with a higher acceptor doping density in the HTL, namely, $N_{A,PTAA} = 1x10^{17}$ cm^{-3} for the opaque cell and $N_{A,PTAA} = 1.5x10^{17}$ cm^{-3} for the semi-transparent cell. The derived J-V characteristics are depicted in Supplementary Fig. 20b), where the cyan solid and dashed lines represent respectively the J-V characteristics of the opaque reference and optimized devices, while the green solid and dashed lines depict the J-V curves of the semi-transparent reference and optimized devices, respectively. We can see that V_{OC} value of the reference device is significantly increased by the unintentional doping while the increment of V_{OC} in the optimized structures is less pronounced, resulting in a smaller improvement of V_{OC} for the semi-transparent cell with respect to the opaque cell, namely, ΔV_{OC} = 32 mV for the opaque contact and ΔV_{OC} = 25 mV for the semi-transparent contact.

The more pronounced effect of unintentional PTAA doping on the reference device can be attributed to the initial less favourable energy level alignment and interfacial contact properties at the perovskite/HTL interface. In the absence of interfacial modifiers such as MX-Cl/4-FPEAI, the reference structure exhibits a higher energy offset for hole extraction and a greater density of interfacial recombination pathways. Increasing the acceptor concentration in PTAA enhances the built-in electric field and improves hole-transporting efficiency, which facilitates charge extraction and reduces interfacial recombination losses, resulting in a notable V_{OC} increase.

In contrast, the optimized device incorporating MX-Cl and 4-FPEAI, already features tailored interfacial energetics and improved chemical passivation. These interlayers contribute to more efficient charge extraction and reduced defect-mediated recombination. As a result, additional doping of PTAA has a comparatively minor effect on V_{OC}, since the contact is already near optimal.

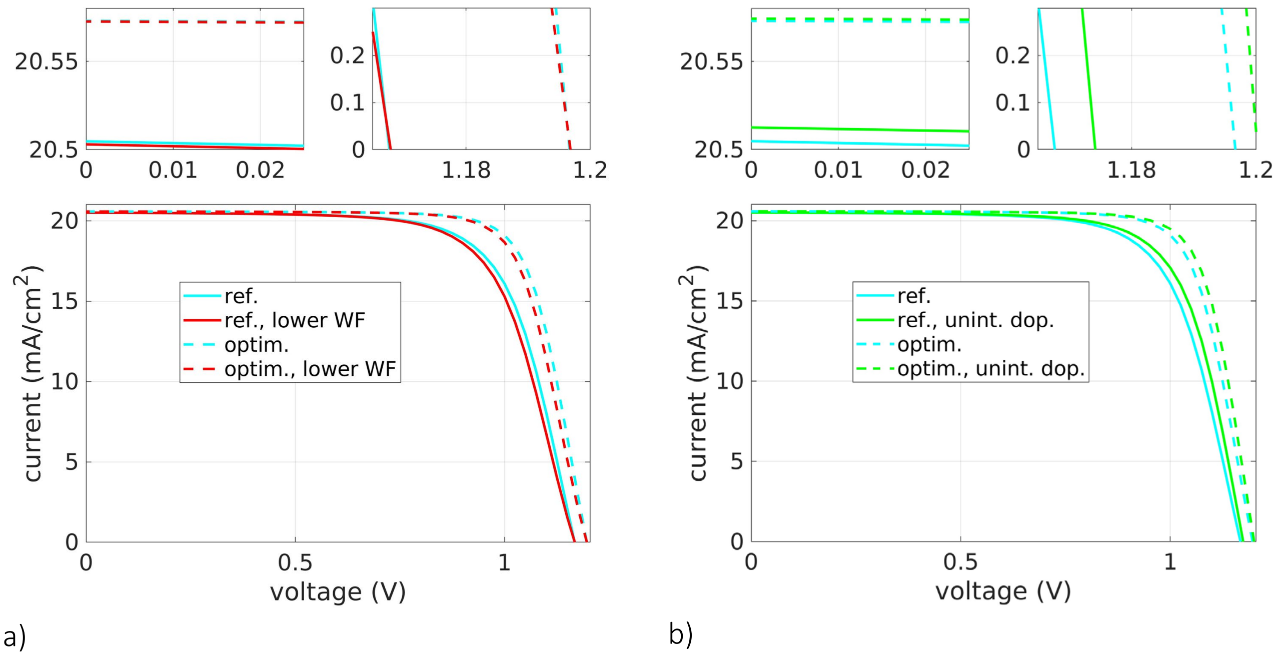

Supplementary Fig. 20: Impact of contact work function and HTL doping on device performance. a) effect of a smaller effective work function value of the p-contact. **b)** effect of a higher acceptor doping density in the hole transport layer.

Discussion on the statistics of ΔV_{OC} and ΔJ_{SC}: we want to point out that, if the top cells are compared, the improvement in V_{OC} of the ST cell is comparable with the value obtained for the opaque cell, namely, ΔV_{OC} = 29 mV for the opaque structure and ΔV_{OC} = 23 mV for the ST structure. Considering the known relation existing between ΔV_{OC} and ΔJ_{SC}, *i.e.*, $V/V_0 \sim \ln(J/J_0)$, the fluctuations in the observed values of ΔV_{OC} are related to the fluctuations in the increment of J_{SC}, as suggested by the figure below (Supplementary Fig. 21), where the distributions of the improvement of V_{OC} (Supplementary Fig. 21a) and J_{SC} (Supplementary Fig. 21b) features for the opaque and ST structures are shown. So, in our opinion, it may be misleading the comparison of the average improvement in V_{OC}, since the width of the ΔV_{OC} distribution of the ST device is larger with

respect to the opaque device, due to the related broader ΔJ_{SC} distribution, and the specific correlation between ΔV_{OC} and ΔJ_{SC} would be lost. We think that, at this stage, the specific comparison between the top cells in the two sets of samples is more reliable. However, this analysis highlights the importance of achieving major control on the deposition process of the ITO layer. Such a strategy could be addressed in our future work.

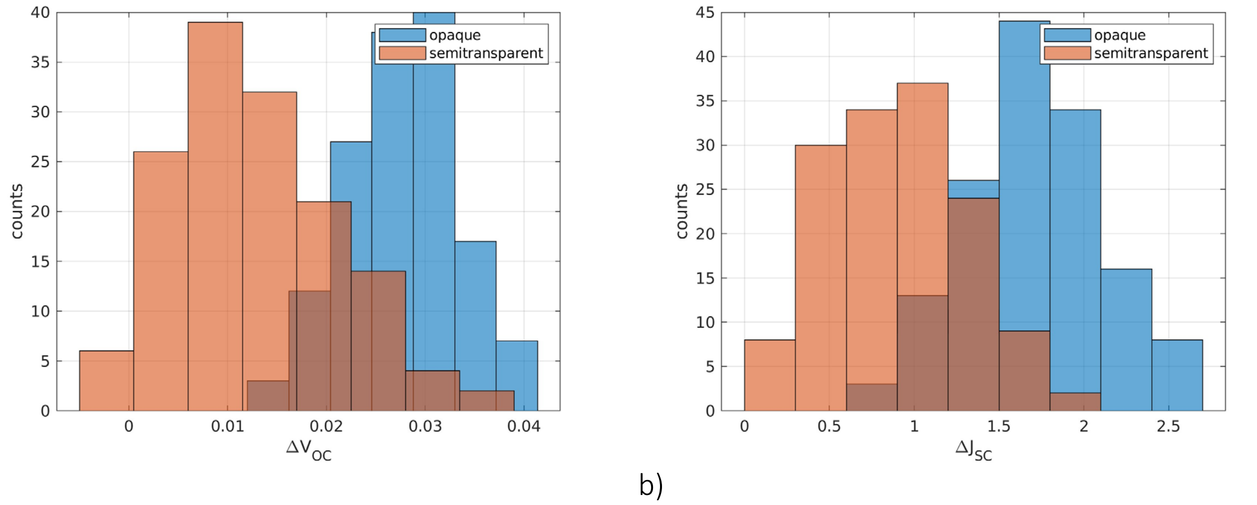

Supplementary Fig. 21: Correlation between ΔV_{OC} and ΔJ_{SC} in opaque and ST devices. Distribution of **a)** $\Delta V_{OC} = V_{OC\_optimized} - V_{OC\_eference}$ and **b)** $\Delta J_{SC} = J_{SC\_optimized} - J_{SC\_reference}$ for the opaque (blue) and semitransparent (red) structures.

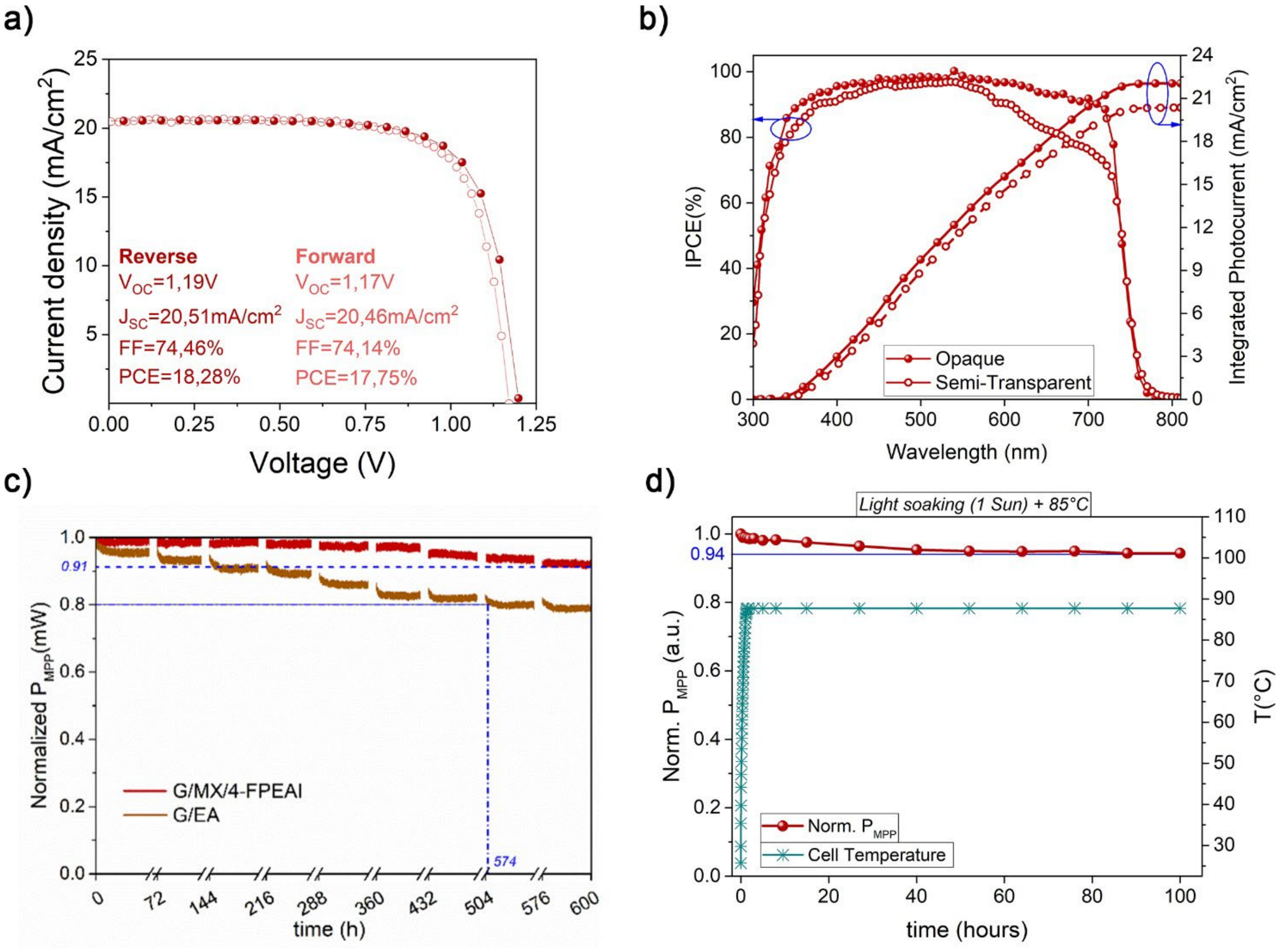

Supplementary Fig. 22: Performance and stability of optimized semi-transparent devices. a) J-V curve in forward and reverse scan for the best-performing 2D material-engineered ST-PSC (G/MX/4-FPEAI structure); **b)** IPCE spectra with the integrated photocurrent density (Integrated J_{SC}) related to the best efficient optimized cell (G/MX/4-FPEAI) for both opaque and ST proposed device structure. **c)** Indoor photo-stability under MPP tracking of G/EA and G/MX/4-FPEAI semi-transparent encapsulated cells upon exposure to cycled light (ISOS-LC-1); **d)** Normalised power at maximum power point (P_{MPP}) continuously acquired during a light soaking test performed under a solar simulator class B @ 1 Sun (circle dotted red curve). Substrate temperature has been monitored by a thermocouple (star dotted dark cyan curve) fixed on the device top surface, showing an increasing value up to 87°C during the first stress test hour, while retaining a constant value during the rest of the accelerated test.

External quantum efficiency (IPCE) spectra of opaque and semi-transparent perovskite solar cells. The opaque device exhibits a higher IPCE across the entire spectral range, particularly between 580 and 730 nm, indicating enhanced photon harvesting due to increased optical path length and efficient photon recycling. In contrast, the semi-transparent cell, incorporating a gold/ITO rear electrode, shows a reduced IPCE response in this region, consistent with optical losses from the transparent electrode and limited back reflection. This reduction contributes to J_{SC} loss of approximately 2.0 mA/cm² in case of the optimized G/MX/4-FPEAI device structure. (Supplementary Fig. 22b)

Indoor photo-stability under MPP tracking of semi-transparent encapsulated cells upon exposure to cycled light. As recently discussed in literature, the most effective way to perform a reliable stability analysis consists in light cycling test at constant temperature, representing a good trade-off between simplicity of execution in lab conditions while taking into account phenomena observed only in case of outdoor testing (that cannot be considered in case of constant light experiments).[15] Indeed, transient behaviour occurs in PSCs as a result of the coexistence of several dynamics with characteristic times spanning from time scales of seconds to hours. Thus, day–night cycling of devices can consider such slow dynamics as they occur on similar timescales. Following this idea, we performed a light–dark cycling test (Supplementary Fig. 22c) as recommended by the ISOS-LC-1 protocol, exposing the cells to simulated sunlight turned on and off with cycle periods of 24 h and duty cycles (light:dark) of 1:2, mimic an accelerated diurnal sun cycle (the solar cell is maintained at ambient conditions while the temperature is monitored and RH is maintained at 30%).[16] The recorded maximum power (P_{MPP}) is reported in Supplementary Fig. 7e for both G/EA and fully optimized G/MX/4-FPEAI semi-transparent cells, normalized at the initial value. The combined stabilization effect of the 4-FPEAI based SGP together and the presence of MX-Cl results in a T_{91}=600 hours vs a T_{80}=574 hours of the untreated device, demonstrating the superior stability (T_{91} 6.5 times larger) of device employing the proposed perovskite field-effect junction approach. Despite more in-depth analyses are required to elucidate the role of MX-Cl and 4-FPEAI SGP, we can confidently ascribe the improved stability to a synergistic effect of the two treatments. On one side, the addition of MX-Cl resulted in a perovskite film with improved crystallinity eventually reducing lattice distortion, lowering defect density and minimizing trap states, as demonstrated by XRD, transient PL and SEM characterizations. This, combined with the enlarged perovskite domains and mitigated grain boundary-induced trap states, makes the trapped charge driven degradation less effective,[17] resulting in an overall enlargement of device lifetime. We cannot exclude a side role of the Cl in reducing the tensile strain within the perovskite layer, recently pointed out as one of the main degradation channel when light-dark cycling is applied.[18] On the other side, the phenylalkylammonium compounds as 4-FPEAI has been proven to stabilize the perovskite through suppression of iodide ion migration, likely responsible for metastable perovskite behaviour under light cycling observed for the G/EA device, while passivating the surface defects.[19] In addition, the 4-FPEAI SGP strategy has been already proven to improve the long-term operational stability of the encapsulated devices under the continuous illumination in ambient condition.[13] Thus, the superior light cycling stability observed in the field-effect junction **s**cell can be confidently ascribed to a combined stabilizing effect from 4-FPEAI and MX-Cl, definitively reducing the device metastable behaviour. Although light-cycling test at MPP is widely recognized as a reliable indicator of device operational stability, this test may underestimate the coupled effects of light soaking and temperature, since the typically employed white LED illumination sources maintain device temperature in the 35–40 °C range. To directly account for these limitations, we carried out an additional stability test under a class-B sun simulator (1000

W m^{-2}, see experimental section for further details about the apparatus), simultaneously tracking P_{MPP} and the front-surface temperature. Under this configuration, the cell temperature overcame 85 °C within the first hour, and the device was continuously stressed for 100 hours. As shown in Supplementary Fig. 22d, the perovskite field-effect junction device retained more than 95% of its initial P_{MPP}, demonstrating excellent thermal and photo-operational stability under harsh conditions.

SECTION S.I. 10 Optimization laser ablation parameters for series interconnections in semi-transparent solar modules (ST-PSMs).

In other to achieve series interconnection of the cells in large area modules through the so-called P1-P2-P3 method an UV laser ablation was used and schematically represented in Supplementary Fig. 23a and b. Firstly, the P1 process insulates the bottom TCO electrode of adjacent cells. After deposition of all the layers up to the hole transporting layer (HTL), the P2 step selectively removes the entire layer stack except for the TCO, which allows the series connection with the subsequent cell upon deposition of the top electrode. Finally, the P3 process is applied to separate the just deposited top electrode of subsequent cells. For this process, selecting a stack of layers is highly important to determine the scribing process for the P1-P2-P3 interconnection. In particular, an adjusted scribing process allows to obtain the solar cell structure optimized and described in the main test and was able to effectively fabricate highly efficient semi-transparent perovskite modules (ST-PSMs). The optimization consisted of finding the ideal fluence per pulse, which is the energy of a single laser pulse divided by the area of the laser spot, as well as the ideal pulse overlap (number of pulses scribed by the laser in a fixed path length or time) to remove the layers corresponding to each scribing process without damaging the rest.

P1 scribing of the FTO-coated glass was the easiest since there are no layers underneath the FTO that could be damaged by the laser. The optimum fluence was found to be 70 mJ cm^{-2} $pulse^{-1}$ with a pulse overlap of 2000 (i.e., 2000 pulses were emitted by the laser in one scribing scan) and was able to remove a single FTO line of about 15µm wide. P3 is slightly more challenging because the top ITO electrode needs to be removed without damaging the neighbouring layers with the parasitic heat induced by the laser pulses. However, a good electrical insulation was obtained by imposing a fluence of 30 mJ cm^{-2} $pulse^{-1}$ and a pulse overlap of 65, resulting in a P3 width of about 100 µm.

The main and most complicated step of the optimization was the P2 scribing. P2 should remove all the layers from the PTAA hole transporting layer to the c-TiO_2 hole blocking layer without damaging the FTO back contact. The UV wavelength of 335 nm used by the laser is absorbed by all the layers that compose the solar cell, including the FTO. Therefore, a balance between incomplete stack removal at low fluence values, and FTO back contact damage at high fluence values should be met. We tested a set of 7 fluence values ranging between 10 and 40 mJ cm^{-2} $pulse^{-1}$ with a step of 5 mJ cm^{-2} $pulse^{-1}$ and a set of 20 pulse overlaps ranging from 100 to 2000 with a step of 100. We constructed a matrix with the different P2 scribe parameters assessed on a substrate equipped with all the layers of our optimized solar cell and observed it under the optical microscope as shown on Supplementary Figs. 23c and 23d. The left axis corresponds to increasing fluence values and the bottom axis to increasing pulse overlap values.

It can be noticed that at low pulse overlap values, the different pulses can be distinguished, whereas for higher pulse overlaps, the boundaries between pulses can no longer be seen and a single uniform line is obtained instead. Moreover, as the fluence increases the lines darken, indicating that the scribes are digging down to deeper layers. After analysing the different components of the matrix by mean of confocal microscopy, we determined that the optimum combination of fluence and pulse overlap for which a uniform, linear removal of the stack was obtained without damaging the FTO back contact was 40 mJ cm^{-2} $pulse^{-1}$ and 900.

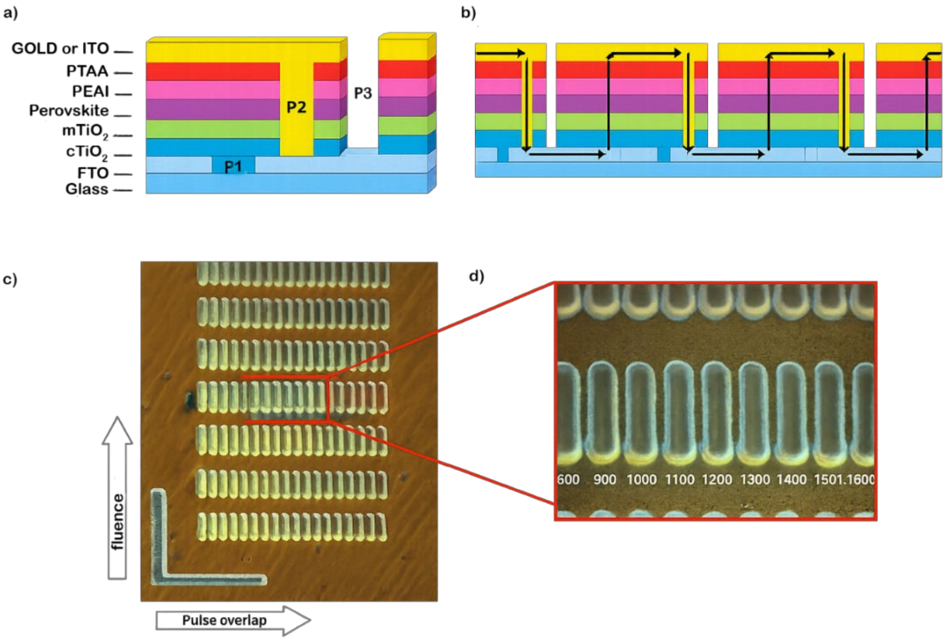

Supplementary Fig. 23: Laser scribing optimization for series interconnection in ST modules. a) Scheme of the used n-i-p cell structure and of the P1-P2-P3 interconnection. **b)** Scheme of the current flow within the series-interconnected cells. **c)** Matrix of the different fluence and pulse overlap combinations for the P2 scribe. **d)** Zoomed-in shot of the 40 mJ cm^{-2} $pulse^{-1}$ fluence part of the matrix focused on pulse overlaps ranging from 800 to 1600.

SECTION S.I. 11 Optimization of ST-PSM layout in term of charge collecting bus-bars.

Another major innovation introduced in the perovskite module layout, consisted in moving one of the current collecting bus-bars from its original position to the top side of the module, by replacing it with a narrower silver finger, in order to minimize the shadowing once stacked atop the Si cell (see module layout schematic reported in the main text in Fig. 5a. In Supplementary Fig. 24 a schematic of the module layout is represented while the I-V characteristics for 2 modules realized with and without the silver finger are reported. In particular, the role of the silver finger in preventing the module FF drop was evident.

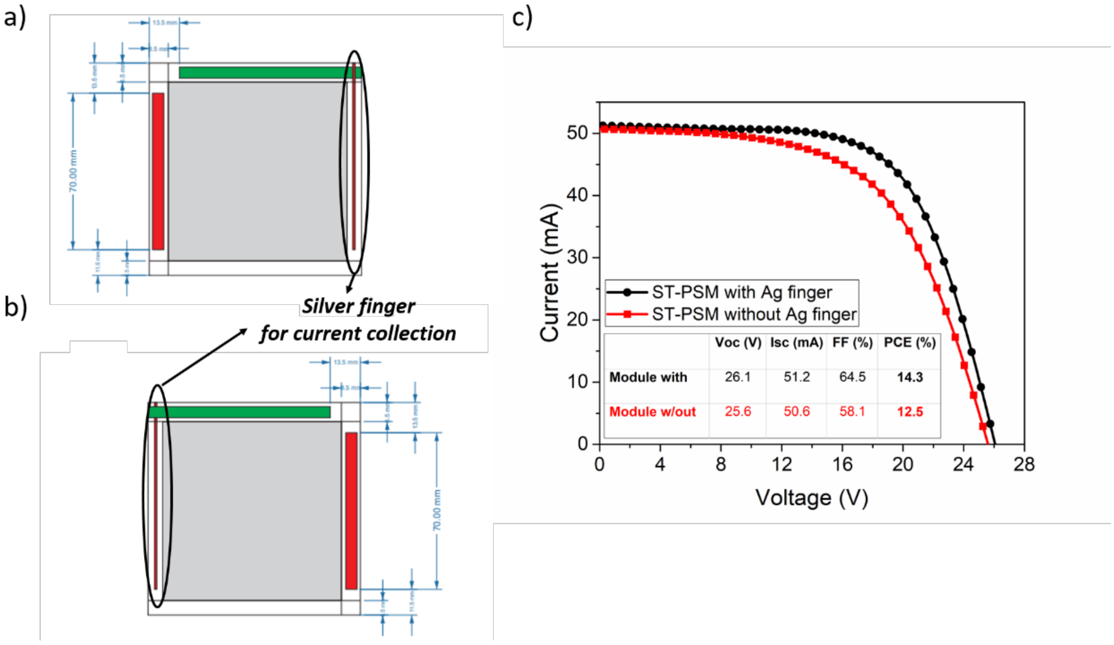

Supplementary Fig. 24: Bus-bar layout optimization for charge collection in perovskite modules. Schematic representation of the electrical connections in a perovskite module (**a-left** and **b-right** configuration in view of covering a M2-sized Si-HJT bottom cell), where the collecting current silver finger was inserted. **c)** I-V characteristics of two ST-PSMs with and without the silver finger.

Cell-to-module performance gap analysis.

To clarify the link between small-area opaque devices and the performance of large-area semi-transparent (ST) modules, we carried out a loss analysis identifying the main contributing factors. The best-performing opaque device (G/MX/4-FPEAI) exhibited a PCE of 22.12%, while the corresponding 60 cm² ST module reached 16.2%, resulting in an absolute performance drop of ~5.96 percentage points.

This gap can be attributed to three main sources:

(i) Optical losses due to the absence of the reflective metal electrode in ST configurations, which reduces photon recycling and light harvesting. To quantify the effect of ST electrode on photovoltaic performance, we compared our optimized opaque device (G/MX/4-FPEAI) with its ST counterpart featuring an Au/ITO top electrode. The opaque device exhibited a PCE of 22.12%, while the ST cell reached 18.3%, under identical illumination and processing conditions (see Supplementary Tab. 7). This ~17.2 % relative efficiency drop (≈3.82 absolute percentage points) can be attributed primarily to optical losses due to the removal of the reflective metal electrode, which otherwise enhances internal light trapping and photon recycling. With the aim to quantify the impact of the ST electrode onto the device photocurrent, we performed IPCE measurements on both the best performing opaque and semi-transparent cell, engineered using the field-effect junction strategy. By computing the integrated current density, we can conclude that the replacement of opaque electrode with a ST one (gold/ITO) contributed to a J_{SC} loss of ~2.0 mA/cm². This is in line with what observed by De Wolf, S. and co-workers,[20,21] which employed optical modelling (Transfer Matrix Method (TMM)) of the device stack to predict a 10–12% of incident photons loss due to front-surface reflection and incomplete absorption in the perovskite because of the reduced optical path length. These factors jointly reduce the short-circuit current density (J_{SC}) by ~ 1.8–2.2 mA/cm². In our case the reflection at the glass substrate is the same for both the ST and opaque devices, concluding that the observed J_{SC} drop can be attributed to the absence of photon recycling in the 580-730 nm region of the visible spectra.

(ii) Electrical losses from the transparent electrode stack (Au/ITO), including increased sheet resistance and contact resistance, resulting in an FF drop of ~6.32%. Nonetheless, the retained high V_{OC} (~1.22 V) and FF (~75%) in the ST cell confirm that our band alignment strategy remains effective under reduced optical confinement.

(iii) Scaling-related effects, including increased surface non-uniformity, edge shunting, and series resistance due to larger interconnection paths, contributing to a further loss in FF of -7.5% moving from the ST-cell to the ST-module. FF loss is the main responsible for the observed drop in PCE moving from ST-cell to ST-modules, since V_{OC} and J_{SC} for each large area cells (LAC, with an active area of 2.5 cm^2) composing the module did not undergo a significant drop (V_{OC_LAC}~ 1.25 V and J_{SC_LAC}~ 20.8 mA/cm^2, see Supplementary Tab. 7)

Despite these inevitable losses, the use of a perovskite field-effect junctionvia Cl-MXene doping and 4-FPEAI passivation proved critical in maintaining high V_{OC} (1.226 V) and FF (67-69%) at the module level. This highlights the effectiveness of the interface and band alignment engineering in mitigating efficiency degradation upon scaling and semi-transparency.

Supplementary Tab. 7: Electrical parameters for best performing opaque cell, ST cell and ST module.

Device Type	Area (cm²)	PCE (%)	V_{OC} (V)	J_{SC} (mA/cm²)	FF (%)
Opaque cell (G/MX/4-FPEAI)	0.1	22.12	1.21	22.5	81.82
ST cell (Au/ITO)	0.1	18.3	1.22	20.5	75.5
	**Area (cm²)**	**PCE (%)**	**V_{OC} (V)**	**I_{SC} (mA)**	**FF (%)**
ST module	60.0	16.16	26.98	52.7	67.9

SECTION S.I. 12 Lamination Procedure for 2D material engineered tandem panel (DEM1 and DEM2) and outdoor whether condition for the preliminary I-V measurement in Rome (Italy).

In the optics to reach commercialization, only sealing materials and components industrially ready has been considered. Lamination materials selection took into account both chemical and processing temperature compatibility with the as-developed tandem technology.
The following set of substrates and materials are identified as for the proposed tandem panel lamination:

- Front sheet: ultra-clear 4 mm thick glass tempered to give robustness to full panel keeping a good transmission rate;
- Back sheet: Polymeric transparent foil 150 um thick from 3M with water vapor barrier layer integrated (WVTR < 6 x 10-5 $g/m^2/day$) with <90% transparency (400–1400 nm);
- Primary sealer: Ionomer foils from Juraplast processable at temperature between 120°C and 150°C;
- Edge sealer: Butyl based cordons from H.B.Fuller. processable at temperature between 110°C and 140°C with WVTR < 0.03 $g/m^2/day$;
- Interconnections: charge collector tapes from 3M + tabbing ribbons;
- Encapsulation procedure: hot vacuum lamination.

Tandem panels are realized using the following configurations:

- Si-HJT: 4 cells connected in series; each cell is standard M2 size (156.75 mm X 156.75 mm X 0.2 mm);
- Perovskite (PSK): 16 modules connected in parallel, each module is 95 mm X 95 mm X 2.3 mm (each Si-HJT cell is covered by 4 PSK modules as reported in Fig. 5b in the main text).

a) ST-PSM electrical interconnections

16 PSMs have been electrically connected in parallel to form the ST perovskite top panel, as depicted in Supplementary Fig. 25. Long tabbing ribbons (silver and green lines in the schematic) have been used and fixed on single modules bus bars (red and black pads in the schematic) via charge collector tapes with the addition of paintable silver paste to increase contact surface area. Extra dead area (blue coloured area in the schematic) all around the PSK modules have been added to avoid tabbing ribbon passing on the top of PSK back electrode with the consequent risk of shunts and shadowing of below Si-HJT cells.

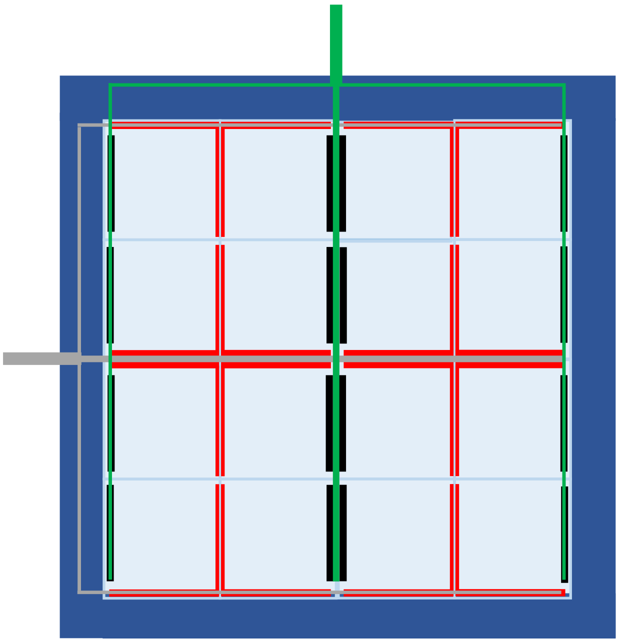

Supplementary Fig. 25: Electrical interconnection scheme of semi-transparent perovskite modules. The schematic of the ST-PSM electrical connection, in particular, the extra dead area is coloured in blue, the tabbing ribbon are highlighted by silver and green lines while positive and negatives contact are represented by the red and dark pads, respectively.

b) Si-HJT cell electrical interconnections

The Si-HJT cells without tabbing ribbons is employed to allow the use of Si-HJT cells 4T tandem architecture. In this case, charger collector tapes from 3M have been used. To avoid vertical shunts risks between Si-HJT contacts and PSK modules ones, a 6 mm width charge collector tapes with an insulator layer integrated on the top was used. The chosen of such width was forced considering the much higher Si-HJT I_{sc} respect to the PSK one, a smaller charge collector tape would have caused a drop in the Si-HJT FF (high contact resistance). Whilst, with 6 mm width tape the Si-HJT FF, under filtered spectrum, is comparable with Si-HJT one at full sun with industrially connected charger collectors using specific electric conductive adhesives (ECAs) and metal tabbing ribbon.

c) All-in-one tandem panel encapsulation

The lamination strategy employed to laminate the 2D-material engineered panels consists in a hot vacuum lamination process following detailed:

a) External substrate 4 mm ultra clear glass;
b) 100 µm ionomer foil as primary sealer;
c) PSK modules positioning and interconnections (see Supplementary Fig. 26a);
d) 400 µm ionomer foil as insulator;
e) Si-HJT cells interconnections and positioning (see Supplementary Fig. 26b);
f) 300 µm ionomer foil as primary sealer;
g) Edge sealer placed all around;
h) Transparent polymeric back sheet with barrier layer;
i) Hot vacuum lamination procedure performed at 120°C, process considering rump up-steady time-rump down it takes about 45 min. The applied pressure 1Bar (see Supplementary Fig. 26c).

The as-described procedure allowed to obtain a “monolithically integrated” tandem device with “one-step” sealing procedure.

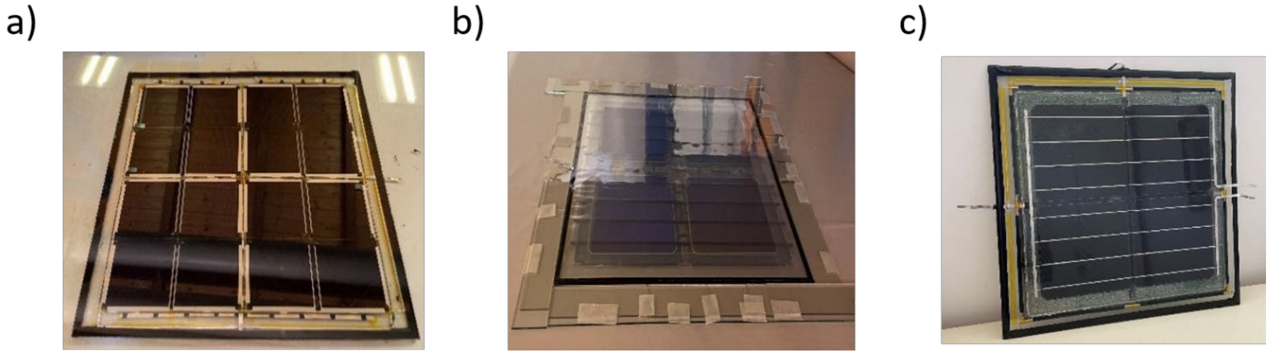

Supplementary Fig. 26: One-step lamination process of 4T perovskite/silicon tandem panels. 4T tandem panel lamination in all-in-one step procedure: **a)** ST-PSM sorting and electrical connections; **b)** Si-HJT cell sorting and electrical connections + deposition atop of the ST ionomer foil as insulator; **c)** final realized 2D material-engineered tandem panel from the back side.

The as-produced DEM2 has been measured outdoor in Rome (41.85371; 12.63508) prior to be shipped in Crete Island for the outdoor stability test. Both ST-perovskite top panel and Si-HJT were measured outdoor on 6th of August 2023 when the irradiance level reach 1000 W/m^2. Irradiance and temperature plots for the whole day at earth suface are reported in Supplementary Fig. 27.

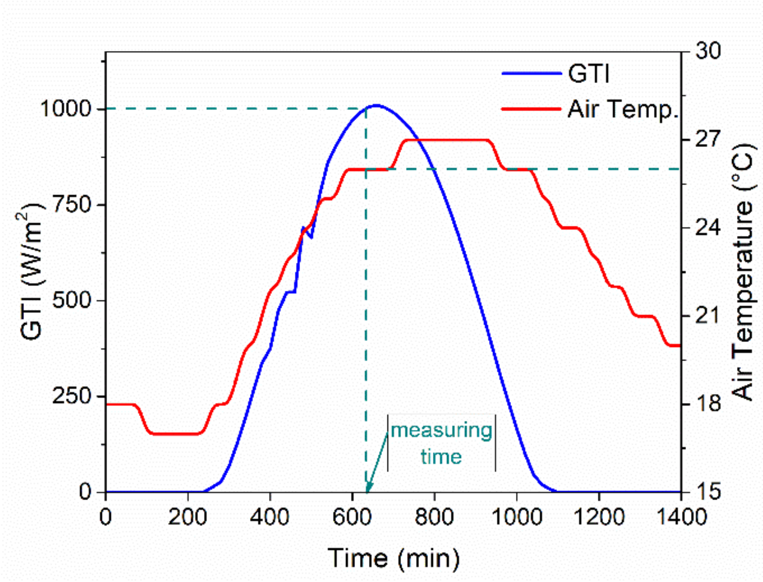

Supplementary Fig. 27: **Outdoor irradiance and temperature during tandem panel testing**. Irradiance (GTI) and air temperature in Rome (41.85371; 12.63508) on 6th of Agust 2023 when measurement on DEM2 have been carried out.

SECTION S.I. 13 Realization and characterization of the stand-alone Si-HJT module (DEM3) as reference single-junction technology.

With the aim to evaluate the performance improvement when moving from the Si-HJT single junction technology to the tandem perovskite/Si-HJT technology, we realized a stand-alone Si-HJT module composed by 4 series connected cells laminated between a front-glass (the same used in the realization of DEM2) and a transparent polymeric back sheet. The lamination strategy employed to laminate the DEM3 is following detailed:

a) External substrate 4 mm ultra clear glass;
b) 100 µm ionomer foil as primary sealer;
c) Si-HJT cells interconnections and positioning;
d) 300 µm ionomer foil as primary sealer;
e) Edge sealer placed all around;
f) Transparent polymeric back sheet with barrier layer;
g) Hot vacuum lamination procedure performed at 120°C, process considering rump up-steady time-rump down it takes about 45 min. The applied pressure 1Bar.

In this way, we fabricated a Si-HJT module having the same electrical connections and same lamination process as those employed for Si bottom module composing the tandem all-in-one DEM2. In the as-realized DEM3, the cell stringing process is manually realized using 6 mm width charger collector tapes from 3M. Conversely, in an industrially relevant production environment, Si-HJT cells are usually stringed and laminated using industrial tools (such as an industrial stringing machine), material specifically design for Si-HJT lamination (such as external glass texturized 3 mm thick, polyolefin foil) and higher temperature hot vacuum lamination procedure (performed at 180°C). All these aspects strongly affect the final Si-HJT module performance. Indeed, when considering the lab-produced module (DEM3) it showed maximum PCE of 16.7% once tested at STC while the Si-HJT realized in the 3SUN industrial facilities showed PCE approaching 18.5% (see Supplementary Tab. 8). The main PCE losses at 1SUN conditions in the case of lab-made DEM3 have to be imputed to series path introduced during the manual cell stringing procedure, eventually penalizing FF and I_{SC}. Conversely, when the irradiance is set to 400 W/m^2 (nearby the irradiance experienced from a filtered Si-HJT module in a tandem panel) the FF gap between the lab-made DEM3 and the 3SUN demonstrator is strongly reduced with respect to the case of 1 SUN irradiation, leading to a PCE gap of around 1 percentage point. Indeed, the lower generated current under reduced irradiance level minimizes the impact of the series resistive path introduced in case of a manual cell stringing process, leading to a more comparable

performance among the lab and industry made Si-HJT module.

Supplementary Tab. 8: **Electrical parameters for both lab-made and 3SUN made Si-HJT module (4 series connected cells) in case of 1SUN and 400 W/m^2 irradiance level.**

Module (4 cells)	V_{oc} (V)	I_{sc} (A)	FF (%)	PCE (%)
**Si-HJT (lab-made) @ STC**	2.86	8.18	57.57	16.71
**Si-HJT (3SUN) @ STC**	2.81	9.16	74.8	18.4
**Si-HJT (lab-made) @ 400 W/m^2**	2.76	3.26	69.8	7.29
**Si-HJT (3SUN) @ 400 W/m^2**	2.81	3.66	77.5	8.33

Thus, for a fair comparison between the proposed 2D materials-engineered tandem panel (DEM2) and the Si-HJT based single junction technology, DEM2 and DEM3 should be compared. From Supplementary Tab. 7, we can estimate an overall PCE increase of 2.74 percentage points when moving from the Si-HJT to tandem technology.

SECTION S.I. 14 Indoor and outdoor characterizations of DEM2 (performed in Heraklion, Crete Island)

Temperature is one of the most important parameters affecting both the electrical properties and the perovskite structure itself[39] due to the intrinsic phenomena occurring in the perovskite. In order to study the behavior of the 4T tandem DEM2, indoor temperature experiments have been performed, specifically designed to eliminate or keep constant some of the many parameters that are constantly changing under outdoor conditions.

In an environmentally controlled room, a six halogen lamps array was placed so that DEM2 block was sufficiently and uniformly irradiated. The irradiance was measured with a CMP3 Kipp & Zonen Pyranometer with sensitivity 9.69x10-6 V/W*m^{-2} on the perovskite top panel, and the irradiance value was 500 W/m^2. A dehumidifier was always in operation in order to keep the humidity at a fixed level (50± 5 %), although it is believed that the humidity cannot affect the panels thanks to the proper lamination ad-hoc developed in this work.

In this way, the only variable parameter is the temperature, and thus it can be observed how the electrical properties of the perovskite top panel change with the gradual increase of the temperature. The DEM2 was placed opposite the lamp array, and a silicone rubber patch thermocouple sensor was placed on the back to monitor the temperature rise. As the temperature increased, the value of the electrical parameters was recorded for each degree Celsius in the range of 35-55 °C. This is the usual operating temperatures range of outdoor systems in Crete. Then, with the help of a cooling system, the gradual cooling of the device under test began with the corresponding recording of the voltage values per degree Celsius. It is worth to note that the voltage temperature dependency for the ST-perovskite top panel was measured to be around -0.3635 %/^{o}C, that is very similar to the one reported in our previous work for the perovskite-based opaque panels.[22]

In Supplementary Tab. 9, the temperature dependence in perovskite and in silicon cell of the tandem 2D material-engineered DEM2 is summarized.

Supplementary Tab. 9: Temperature dependence of perovskite module and silicon cell of tandem DEM2 panel.

	V_{oc} temperature dependence (% /°C)	I_{sc} temperature dependence (% /°C)	P_{max} temperature dependence (% /°C)
**Perovskite top panel**	-0.36	-0.30	-0.35
**HJT-Si bottom module**	-0.18	0.035	-0.25

In addition, the dependence of irradiation in both perovskite module and silicon module of the 4T tandem panel is reported in Supplementary Fig. 28 panel a and b respectively. Each electrical parameter has been normalized at the maximum value. For both top panel and bottom module, the total I_{SC} increased almost linearly with the irradiance level, whereas V_{OC} data indicate a logarithmic dependence, which is expected for both PSCs and Si-HJT cells operating at light intensities >10 W m^{-2}.[23,24] The maximum power (P_{MPP}) followed the irradiance almost linearly (Supplementary Fig. 28) as a consequence of the trends observed for the other PV metrics.

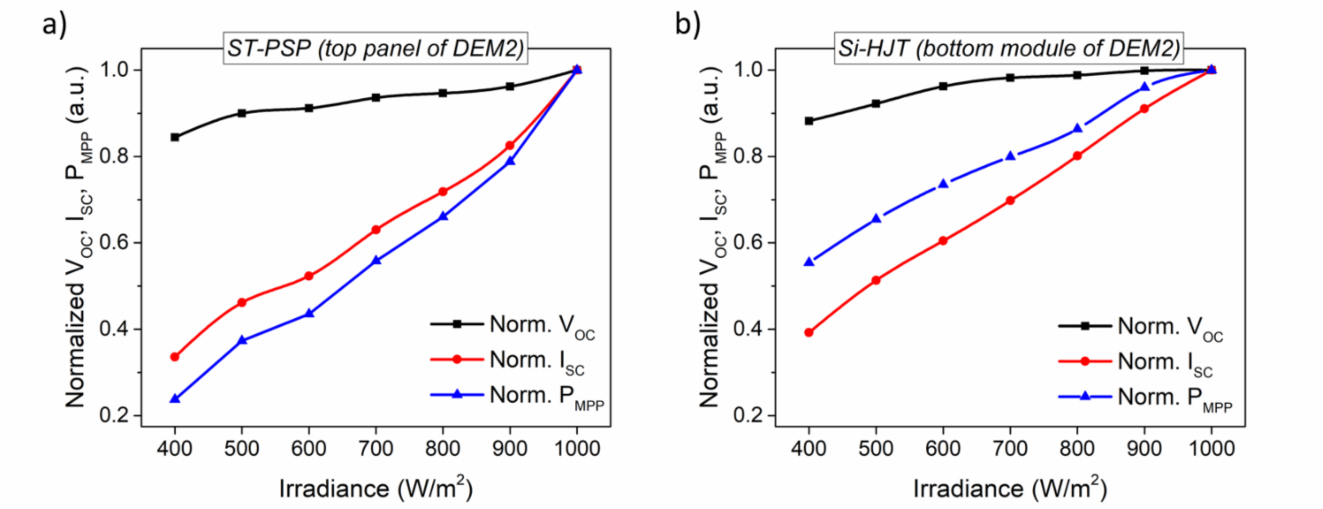

Supplementary Fig. 28: Irradiance dependence of electrical parameters in tandem sub-cells. Irradiance dependency of the normalized electrical parameters (V_{OC}, I_{SC}, P_{MPP}) for both **a)** top perovskite panel and **b)** Si-HJT bottom module forming the DEM2 during nominal operational conditions (NOCT) in a clear day in Solar Farm facilities in Heraklion.

The prolonged outdoor testing was performed on DEM2 panel at open circuit condition, following the ISOS-O-2 protocol. Thus, the panel was not "continuously monitored" in a strict sense but was left mounted on the rack in the operative position at open-circuit and measured once per month. The parameters reported in Fig.7f were extracted from the I-V curves, acquired for the perovskite top panel after a stabilization time of 10 minutes, since we observed an increase in the delivered power till reaching a stabilized value after 10 minutes (see Supplementary Fig. 29a). Since the outdoor characterizations are typically subjected to the instantaneous variation of the irradiance and the panel temperature, we selected a clear sky day in the first half of the month performing the measurements when the irradiance was supposed to be maximum (at 12:30 am). The irradiance daily curves for Hellenic Mediterranean University site in Heraklion (35.31959; 25.10244) recorded during the day when the outdoor I-V curve were acquired, are reported in Supplementary Fig. 29b. Once acquired the I-V curves, the extracted electrical parameters were normalized in STC (1000 W/m^2 irradiance and 25°C temperature) considering i) the panel temperature and the irradiance values recorded

at the measurement time (at 12:30 am, see values reported in Supplementary Tab. 10) ii) both temperature and irradiance dependency of the main electrical parameters reported in Supplementary Fig. 28.

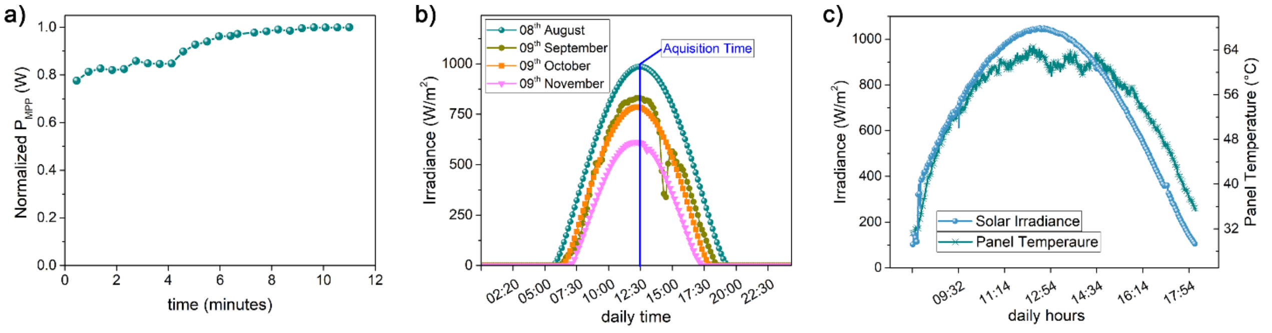

Fig. 29: Stabilization behaviour and outdoor conditions during long-term testing. a) Normalized power at maximum power point (P_{MPP}) stabilization prior the acquisition of the monthly I-V curves for the DEM2 left at V_{OC} in outdoor conditions; **b)** daily irradiance for the days selected during the outdoor test for the acquisition of the I-V curves. **c)** Top-surface temperature of the tandem panel recorded under real operating conditions between August and November 2023. Even during peak irradiance at midday under clear-sky conditions, the module temperature did not exceed ~65 °C, providing a realistic reference for assessing the operational thermal load experienced by the devices.

Supplementary Tab. 10: Photovoltaic parameters of the perovskite top panel (DEM2) during the three-month outdoor stability test and quantitative evaluation of performance degradation over time. The electrical data were obtained by acquiring the I-V characteristics in outdoor condition in selected days (one per each stress month; panel temperature and irradiance values at the I-V curve acquisition time are reported in the first two columns) at 12:30 am and subsequently normalized in STC (1000 W/m^2 irradiance and 25°C temperature).

	Panel T (°C)	Irradiance (W/m^2)	V_{OC}(V)	I_{SC}(A)	FF(%)	Power(W)
**8th August 2023**	61.3	986	26.4	0.76	52.2	10.5
**9th September 2023**	53.9	829	26.6	0.76	51.6	10.5
**9th October 2023**	50.4	782	27.1	0.75	50.9	10.4
**9th November 2023**	43.5	608	26.6	0.74	50.2	10.0
**Total degradation over three months**			0.76%	-2.63	-3.83%	-4.76%

Supplementary Tab. 10 summarizes the photovoltaic parameters of the perovskite top panel (DEM2) during outdoor testing from August to November 2023. The results show a gradual decrease in FF (≈-3.1%) and I_{SC} (≈-2.6%) over the three-month period, while V_{OC} remains nearly constant. The overall power output decreases by ≈-4.8%, corresponding to a retained performance of about 95% of the initial value.

Finally, we created a test setup to identify the bifaciality factor that panels can achieve by rotating the tandem panels into the sun and put the silicon module on top side. With the last measurement we could identify the energy produced from the back silicon module in any ground surface, with the knowledge of the albedo of the installation surface. In Supplementary Tab. 11 the most used ground surfaces for installations of PV parks are presented.

Supplementary Tab. 11: Albedo Irradiance in different ground surface types

Surface Type	Albedo
**Green Field Grass**	23%
**Concrete**	20%
**White Painted concrete**	60-80%
**White Gravel**	27%
**White roofing metal**	56%
**Membrane Light Grey**	62%
**Membrane White**	>80%
**Snow**	80%

Supplementary References

(1) Kamysbayev, V.; Filatov, A. S.; Hu, H.; Rui, X.; Lagunas, F.; Wang, D.; Klie, R. F.; Talapin, D. V. Covalent Surface Modifications and Superconductivity of Two-Dimensional Metal Carbide MXenes. *Science (80-.).* **2020**, *369* (6506), 979–983. https://doi.org/10.1126/science.aba8311.

(2) Benchakar, M.; Loupias, L.; Garnero, C.; Bilyk, T.; Morais, C.; Canaff, C.; Guignard, N.; Morisset, S.; Pazniak, H.; Hurand, S.; et al. One MAX Phase, Different MXenes: A Guideline to Understand the Crucial Role of Etching Conditions on Ti3C2Tx Surface Chemistry. *Appl. Surf. Sci.* **2020**, *530* (July). https://doi.org/10.1016/j.apsusc.2020.147209.

(3) Li, M.; Lu, J.; Luo, K.; Li, Y.; Chang, K.; Chen, K.; Zhou, J.; Rosen, J.; Hultman, L.; Eklund, P.; et al. Element Replacement Approach by Reaction with Lewis Acidic Molten Salts to Synthesize Nanolaminated MAX Phases and MXenes. *J. Am. Chem. Soc.* **2019**, *141* (11), 4730–4737. https://doi.org/10.1021/jacs.9b00574.

(4) Xu, G.; Xue, R.; Chen, W.; Zhang, J.; Zhang, M.; Chen, H.; Cui, C.; Li, H.; Li, Y.; Li, Y. New Strategy for Two-Step Sequential Deposition: Incorporation of Hydrophilic Fullerene in Second Precursor for High-Performance P_I_N Planar Perovskite Solar Cells. *Adv. Energy Mater.* **2018**, *8*, 1703054. https://doi.org/10.1002/aenm.201703054.

(5) Liu, X. X.; Zhang, Z.; Jiang, J.; Tian, C.; Wang, X.; Wang, L.; Zhang, Z.; Wu, X.; Zheng, Y.; Liang, J.; et al. Chlorine-Terminated MXene Quantum Dots for Improving Crystallinity and Moisture Stability in High-Performance Perovskite Solar Cells. *Chem. Eng. J.* **2022**, *432* (December 2021), 134382. https://doi.org/10.1016/j.cej.2021.134382.

(6) Zhu, T.; Su, J.; Labat, F.; Ciofini, I.; Pauporte, T. Interfacial Engineering through Chloride-Functionalized Self- Assembled Monolayers for High-Performance Perovskite Solar Cells ́ e R. *Appl. Mater. Interfaces* **2020**, *12*, 744–752. https://doi.org/10.1021/acsami.9b18034.

(7) Hang, P.; Xie, J.; Li, G.; Wang, Y.; Fang, D.; Yao, Y.; Xie, D.; Cui, C.; Yan, K.; Xu, J.; et al. An Interlayer with Strong Pb-Cl Bond Delivers Ultraviolet-Filter-Free , Efficient , and Photostable Perovskite Solar Cells An Interlayer with Strong Pb-Cl Bond Delivers Ultraviolet-Filter-Free , Efficient , and Photostable Perovskite Solar Cells. *iScience* **2019**, *21*, 217–227. https://doi.org/10.1016/j.isci.2019.10.021.

(8) Wang, Y.; Wu, T.; Barbaud, J.; Kong, W.; Cui, D.; Chen, H.; Yang, X.; Han, L. Stabilizing Heterostructures of Soft Perovskite Semiconductors. *Science (80-.).* **2019**, *365* (August), 687–691.

https://doi.org/epdf/10.1126/science.aax8018.

(9) Lopez-Varo, P.; Jiménez-Tejada, J. A.; García-Rosell, M.; Ravishankar, S.; Garcia-Belmonte, G.; Bisquert, J.; Almora, O. Device Physics of Hybrid Perovskite Solar Cells: Theory and Experiment. *Adv. Energy Mater.* **2018**, *8* (14). https://doi.org/10.1002/aenm.201702772.

(10) Carron, R.; Andres, C.; Avancini, E.; Feurer, T.; Nishiwaki, S.; Pisoni, S.; Fu, F.; Lingg, M.; Romanyuk, Y. E.; Buecheler, S.; et al. Bandgap of Thin Film Solar Cell Absorbers : A Comparison of Various Determination Methods. *Thin Solid Films* **2019**, *669* (November 2018), 482–486. https://doi.org/10.1016/j.tsf.2018.11.017.

(11) Ummadisingu, A.; Meloni, S.; Mattoni, A.; Tress, W.; Grätzel, M. Crystal-Size-Induced Band Gap Tuning in Perovskite Films. *Angew. Chemie - Int. Ed.* **2021**, *60* (39), 21368–21376. https://doi.org/10.1002/anie.202106394.

(12) Agresti, A.; Pazniak, A.; Pescetelli, S.; Di Vito, A.; Rossi, D.; Pecchia, A.; Auf der Maur, M.; Liedl, A.; Larciprete, R.; Kuznetsov, D. V.; et al. Titanium-Carbide MXenes for Work Function and Interface Engineering in Perovskite Solar Cells. *Nat. Mater.* **2019**, *18*, 1228–1234. https://doi.org/10.1038/s41563-019-0478-1.

(13) Yan, N.; Gao, Y.; Yang, J.; Fang, Z.; Feng, J.; Wu, X.; Chen, T.; Liu, S. F. Wide-Bandgap Perovskite Solar Cell Using a Fluoride-Assisted Surface Gradient Passivation Strategy. *Angew. Chem. Int. Ed.* **2023**, *62*, e202216668. https://doi.org/10.1002/anie.202216668.

(14) Sutanto, A. A.; Caprioglio, P.; Drigo, N.; Hofstetter, Y. J.; Garcia-Benito, I.; Queloz, V. I. E.; Neher, D.; Nazeeruddin, M. K.; Stolterfoht, M.; Vaynzof, Y.; et al. 2D / 3D Perovskite Engineering Eliminates Interfacial Recombination Losses in Hybrid Perovskite Solar Cells Eliminates Interfacial Recombination Losses in Hybrid Perovskite Solar Cells. *Chem* **2021**, *7*, 1903–1916. https://doi.org/10.1016/j.chempr.2021.04.002.

(15) Khenkin, M.; Köbler, H.; Remec, M.; Roy, R.; Erdil, U.; Li, J.; Phung, N.; Adwan, G.; Paramasivam, G.; Emery, Q.; et al. Light Cycling as a Key to Understanding the Outdoor Behaviour of Perovskite Solar Cells. *Energy Environ. Sci.* **2023**, *17* (2), 602–610. https://doi.org/10.1039/d3ee03508e.

(16) Khenkin, M. V.; Katz, E. A.; Abate, A.; Bardizza, G.; Berry, J. J.; Brabec, C.; Brunetti, F.; Bulović, V.; Burlingame, Q.; Di Carlo, A.; et al. Consensus Statement for Stability Assessment and Reporting for Perovskite Photovoltaics Based on ISOS Procedures. *Nat. Energy* **2020**, *5* (1), 35–49. https://doi.org/10.1038/s41560-019-0529-5.

(17) Ahn, N.; Kwak, K.; Jang, M. S.; Yoon, H.; Lee, B. Y.; Lee, J.; Pikhitsa, P. V; Byun, J.; Choi, M. Trapped Charge-Driven Degradation of Perovskite Solar Cells. *Nat. Commun.* **2016**, *8* (May), 1–9. https://doi.org/10.1038/ncomms13422.

(18) Shen, Y.; Zhang, T.; Xu, G.; Steele, J. A.; Chen, X.; Chen, W.; Zheng, G.; Li, J.; Guo, B.; Yang, H.; et al. Strain Regulation Retards Natural Operation Decay of Perovskite Solar Cells. *Nature* **2024**, *635* (8040), 882–889. https://doi.org/10.1038/s41586-024-08161-x.

(19) Guo, Y.; Apergi, S.; Li, N.; Chen, M.; Yin, C.; Yuan, Z.; Gao, F.; Xie, F.; Brocks, G.; Tao, S.; et al. Phenylalkylammonium Passivation Enables Perovskite Light Emitting Diodes with Record High-Radiance Operational Lifetime: The Chain Length Matters. *Nat. Commun.* **2021**, *12* (1). https://doi.org/10.1038/s41467-021-20970-6.

(20) Löper, P.; Moon, S.-J.; Nicolas, S. M. de; Niesen, B.; Ledinsky, M.; Nicolay, S.; Bailat, J.; Yum, J.-H.; Wolf, S. De; Ballif, C. Organic-Inorganic Halide Perovskite / Crystalline Silicon Four-Terminal Tandem Solar Cells Philipp. *Phys. Chem. Chem. Phys.* **2015**, *17*, 1619–1629. https://doi.org/https://doi.org/10.1039/C4CP03788J.

(21) De Wolf, S.; Holovsky, J.; Moon, S. J.; Löper, P.; Niesen, B.; Ledinsky, M.; Haug, F. J.; Yum, J. H.; Ballif,

C. Organometallic Halide Perovskites: Sharp Optical Absorption Edge and Its Relation to Photovoltaic Performance. *J. Phys. Chem. Lett.* **2014**, *5* (6), 1035–1039. https://doi.org/10.1021/jz500279b.

(22) Pescetelli, S.; Agresti, A.; Viskadouros, G.; Razza, S.; Rogdakis, K.; Kalogerakis, I.; Spiliarotis, E.; Leonardi, E.; Mariani, P.; Sorbello, L.; et al. Integration of Two-Dimensional Materials-Based Perovskite Solar Panels into a Stand-Alone Solar Farm. *Nat. Energy* **2022**, *7*, 597–607. https://doi.org/10.1038/s41560-022-01035-4.

(23) Gouda, L.; Gottesman, R.; Ginsburg, A.; Keller, D. A.; Haltzi, E.; Hu, J.; Tirosh, S.; Anderson, A. Y.; Zaban, A.; Boix, P. P. Open Circuit Potential Build-Up in Perovskite Solar Cells from Dark Conditions to 1 Sun. *J. Phys. Chem. Lett.* **2015**, *6*, 4640–4645. https://doi.org/10.1021/acs.jpclett.5b02014.

(24) Ru, X.; Yang, M.; Yin, S.; Wang, Y.; Hong, C.; Peng, F.; Yuan, Y.; Sun, C.; Xue, C.; Qu, M.; et al. Silicon Heterojunction Solar Cells Achieving 26.6% Efficiency on Commercial-Size p-Type Silicon Wafer. *Joule* **2024**, *8* (4), 1092–1104. https://doi.org/10.1016/j.joule.2024.01.015.
