# Supplementary material for: MXene-driven nanoscale field-effect junction for advanced 4-terminal perovskite/silicon tandem solar panels
Source: Nat Commun. 2026 Mar 6;17:3394. doi: 10.1038/s41467-026-70002-4 (PMC13065735; doi:10.1038/s41467-026-70002-4)
Supplement: Supplementary file 2 — Reporting Summary [file 41467_2026_70002_MOESM2_ESM.pdf]

nature portfolio

Corresponding author(s): Prof. Antonio Agresti

Solar Cells Reporting Summary

Nature Portfolio wishes to improve the reproducibility of the work that we publish. This form is intended for publication with all accepted papers reporting the characterization of photovoltaic devices and provides structure for consistency and transparency in reporting. Some list items might not apply to an individual manuscript, but all fields must be completed for clarity.

For further information on Nature Research policies, including our data availability policy, see Authors & Referees.

Experimental design

Please check the following details are reported in the manuscript, and provide a brief description or explanation where applicable.

1. Dimensions

Area of the tested solar cells

☒ Yes
☐ No

Perovskite solar cell: active area 0.1 cm2, perovskite solar module: active area 60 cm2, aperture area 63cm2. These information are reported in main text "Small area cells" section, in "Four terminal tandem panels" section and in Fig. 6 .

Explain why this information is not reported/not relevant.

Method used to determine the device area

☒ Yes
☐ No

A shadow mask is used for small area cells, while the active area of the perovskite modules was defined by laser patterning procedure. This is stated in the main text in both "experimental section" and "Four terminal tandem panels" sections.

Explain why this information is not reported/not relevant.

2. Current-voltage characterization

Current density-voltage (J-V) plots in both forward and backward direction

☒ Yes
☐ No

Current density-voltage (J-V) plots are reported in SI section S.I. 4 and in the main text in "Four terminal tandem panels" section, Fig.6.

Voltage scan conditions

☒ Yes
☐ No

Both reverse and forward I–V scans were performed using a scan rate of 20 mVs-1 for masked cells and 200 mV s−1 for the unmasked large area modules.

Explain why this information is not reported/not relevant.

Test environment

☒ Yes
☐ No

The test conditions are indicated in the main text in the section "Four-terminal tandem panels" and in SI, Section S.I. 12 "Lamination Procedure for 2D material engineered tandem mini-panel (DEM1 and DEM2), outdoor whether condition for the preliminary I-V measurement in Rome (Italy)" and Section S.I. 14 "Indoor and outdoor characterizations of DEM2 (performed in Heraklion, Crete Island)".

Explain why this information is not reported/not relevant.

Protocol for preconditioning of the device before its characterization

☐ Yes
☐ No

The Voc for small area devices was stabilized for 30 seconds prior the J-V characteristic acquisition, following the well-established measurement protocol reported in literature. This information is reported in the "experimental section". The power at maximum power point (PMPP) for tandem panel was stabilized outdoor for 10 minutes prior the I-V curve acquisition, as indicated in Section S.I. 14 I"ndoor and outdoor characterizations of DEM2 (performed in Heraklion, Crete Island)."

Explain why this information is not reported/not relevant.

Stability of the J-V characteristic

☒ Yes
☐ No

Small area cells: the stability of the J-V characteristic was verified with time evolution of the Maximum Power Point (MPP) reported in SI section S.I. 4, Fig. S7e; the long-term stability was assesed by performing light–dark cycling test as recommended by the ISOS-LC-1 protocol, reported in Section S.I. 9, Fig. S22c. Moreover, the combined light (1 Sun) + temperature (85°C) prolonged stress test at MPP (ISOS-L-2) is reported in Fig.S22d. The tandem panel outdoor stability was assesed by ISOS-O-2 protocol in open circuit condition and reported in Fig. 7f of the manuscript and detailed in Section S.I. 14 of SI.

Explain why this information is not reported/not relevant.

3. Hysteresis or any other unusual behaviour

Description of the unusual behaviour observed during the characterization

☒ Yes ☐ No

We reported the hysteresis behaviour and related discussion in SI Section S.I. 4 in Fig. S7, in Fig. S22a and in the main text Fig.6b.

Explain why this information is not reported/not relevant.

Related experimental data

☒ Yes ☐ No

Reported in SI Section S.I. 4. Fig. S7, Fig. S22a and in the main text Fig. 6b.

Explain why this information is not reported/not relevant.

4. Efficiency

External quantum efficiency (EQE) or incident photons to current efficiency (IPCE)

☒ Yes ☐ No

For perovskite opaque cells the EQE is reported in SI in Section S.I. 5 Fig. S9b, for semi-transparent perovskite cells the EQE is reported in SI Section S.I. 9 Fig. S22b, while it is not supported for large area devices.

Explain why this information is not reported/not relevant.

A comparison between the integrated response under the standard reference spectrum and the response measure under the simulator

☒ Yes ☐ No

For perovskite opaque cells the EQE is reported in SI in Section S.I. 5 Fig. S9b, for semi-transparent perovskite cells the EQE is reported in SI Section S.I. 9 Fig. S22b, while it is not supported for large area devices.

Explain why this information is not reported/not relevant.

For tandem solar cells, the bias illumination and bias voltage used for each subcell

☐ Yes ☒ No

Provide a description of the measurement conditions.

Tandem modules and panels are realized in a 4-terminals configuration. Each sub-panel is measured outdoor without any bias illumination or voltage, by darkening the back of the panel in case of zero-albedo condition. Outdoor measurements details are reported in SI sections S.I. 12 and S.I. 14.

5. Calibration

Light source and reference cell or sensor used for the characterization

☒ Yes ☐ No

Current–voltage (I–V) characteristics of unmasked and encapsulated modules were acquired in air by using a solar simulator (ABET Sun 2000, class A) as indicate in "experimental section" in the main text.

Explain why this information is not reported/not relevant.

Confirmation that the reference cell was calibrated and certified

☒ Yes ☐ No

Solar simulator (ABET Sun 2000, class A) calibrated
at AM1.5 and 100 mW cm−2 illumination with a certified reference Si cell (RERA Solutions RR-1002), as indicate in "experimental section" in the main text.

Explain why this information is not reported/not relevant.

Calculation of spectral mismatch between the reference cell and the devices under test

☒ Yes ☐ No

Incident power was measured with a Skye SKS 1110 sensor.
The class of the solar simulator was measured with a BLACK-Comet UV–vis spectrometer, as indicated in the "experimental section" in the main text.

Explain why this information is not reported/not relevant.

6. Mask/aperture

Size of the mask/aperture used during testing

☒ Yes ☐ No

For small area perovskite solar cell the size is 0.1 cm2, while module active area is defined by laser scribe process, as indicate in the main text "Small area cells" section and in "Four-terminal tandem panels" section.

Explain why this information is not reported/not relevant.

Variation of the measured short-circuit current density with the mask/aperture area

☐ Yes ☒ No

Report the difference in the short-circuit current density values measured with the mask and aperture area.

The module active area is fixed by the laser scribing process as described in "Four-terminal tandem panels" section.

7. Performance certification

Identity of the independent certification laboratory that confirmed the photovoltaic performance

☐ Yes ☒ No

Identify the independent certification laboratory.

We did not certify the photovoltaic performance because none record PCE has been reported in this work.

A copy of any certificate(s)

☐ Yes ☒ No

Certificate copies should be provided in the Supplementary information. Please state the supplementary item number.

Explain why this information is not reported/not relevant.

8. Statistics

Number of solar cells tested

☒ Yes
☐ No

Small area cells: statistics for opaque devices reported in the main text Fig. 2, statistics for semi-transparent devices reported in SI Section S.I. 9 Figure S19.
Large area modules: statistics over 150 modules reported in Fig. 7a.

Explain why this information is not reported/not relevant.

Statistical analysis of the device performance

☒ Yes
☐ No

Small area cells: statistics for opaque devices reported in the main text Fig. 2, statistics for semi-transparent devices reported in SI Section S.I. 9 Figure S19.
Large area modules: statistics over 150 modules reported in Fig. 7a.

Explain why this information is not reported/not relevant.

9. Long-term stability analysis

Type of analysis, bias conditions and environmental conditions

☒ Yes
☐ No

For small area devices, the long-term stability was assesed by performing i) light–dark cycling test as recommended by the ISOS-LC-1 protocol, reported in Section S.I. 9, Fig. S22c, ii) combined light (1 Sun)+temperature (85°C) prolonged stress test at MPP (ISOS-L-2), reported in Fig.S22d. The tandem panel outdoor stability was assesed by ISOS-O-2 protocol in open circuit condition and reported in Fig. 7f of the manuscript and detailed in Section S.I. 14 of SI.

Explain why this information is not reported/not relevant.
